# F-box protein Fbx23 acts as a transcriptional coactivator to recognize and activate transcription factor Ace1

**DOI:** 10.1371/journal.pgen.1011539

**Published:** 2025-01-21

**Authors:** Zhongjiao Liu, Kexuan Ma, Panpan Zhang, Siqi Zhang, Xin Song, Yuqi Qin

**Affiliations:** 1 National Glycoengineering Research Center, Shandong University, Qingdao, China; 2 State Key Laboratory of Microbial Technology, Shandong University, Qingdao, China; University of Kent, UNITED KINGDOM OF GREAT BRITAIN AND NORTHERN IRELAND

## Abstract

Protein ubiquitination is usually coupled with proteasomal degradation and is crucial in regulating protein quality. The E3 ubiquitin-protein ligase SCF (Skp1-Cullin-F-box) complex directly recognizes the target substrate via interaction between the F-box protein and the substrate. F-box protein is the determinant of substrate specificity. The limited number of identified ubiquitin ligase-substrate pairs is a major bottleneck in the ubiquitination field. *Penicillium oxalicum* contains many transcription factors, such as BrlA, CreA, XlnR, and Ace1, conserved in filamentous fungi that regulate the fungal development and transcription of (hemi)cellulase genes. Transcription factor Ace1 (also known as SltA) positively correlated with fungal growth and conidiation and negatively correlated with the expression of (hemi)cellulase genes. A ubiquitin ligase-substrate pair, SCF^Fbx23^-Ace1, is identified in *P*. *oxalicum*. Most of PoFbx23 is present in free form within the nucleus. A small portion of PoFbx23 associates with Skp1 to form PoFbx23-Skp1 heterodimer or assembles with the three invariable core components (Skp1, Cul1, and Rbx1) of SCF to form the SCF^Fbx23^ complex. Under glucose signal, PoFbx23 absence (Δ*fbx23*) results in decreased transcription levels of the *brlA* gene which encodes the master regulator for asexual development and six spore pigmentation genes (*abrB*→*abrA*→*aygB*→*arpA*→*arpB*→*albA*) which encode the proteins in the dihydroxynaphthalene-melanin pathway, along with impaired conidiation. Under cellulose signal, transcription levels of (hemi)cellulase genes in the Δ*fbx23* mutant are significantly upregulated. When PoFbx23 is present, PoAce1 exists as a full-length version and several low-molecular-weight degraded versions. PoAce1 has polyubiquitin modification. Deleting the Po*fbx23* gene does not affect Po*ace1* gene transcription but results in the remarkable accumulation of all versions of the PoAce1 protein. Accumulated PoAce1 protein is a dysfunctional form that no longer binds promoters of the target gene, including the cellulase genes *cbh1* and *eg1*, the hemicellulase gene *xyn11A*, and the pigmentation-related gene *abrB*. PoFbx23 acts as a transcriptional coactivator, recognizing and activating PoAce1, allowing the latter to regulate the transcription of target genes with different effects (activating or repressing) under different signals.

## Author summary

Protein degradation controls many cellular and developmental processes. Specific ubiquitin-protein ligase E3 plays essential roles in protein degradation by recognizing the target substrate. F-box protein, the variable component of E3, has been at the center of attention as it is the determinant of substrate specificity. Most F-box proteins in eukaryotes remain “orphans”, i.e., their recognizing substrate has yet not been identified. Fbx23, once an orphan protein, is no longer alone with the identification of the “Fbx23-Ace1” pair. The recognition substrate for the specific F-box protein is relatively conserved in various species. The discovery of the Fbx23-Ace1 pair in *P*. *oxalicum* will help in related research of other filamentous fungi. Many F-box proteins, including Fbx23, are linked to the transcription of genes involved in fungal growth, development, secondary metabolism, and extracellular enzyme synthesis in various fungi. The finding that the Fbx23 acts as a transcriptional coactivator to recognize and activate transcription factor Ace1 provides insight into how F-box protein, a protein incapable of binding DNA, controls transcription through the specific transcription factor as a bridge.

## Introduction

Post-translational modifications of proteins play versatile roles in protein functions ranging from protein degradation to subcellular localization, conformation change, and protein affinity with DNA motifs [1]. Protein ubiquitination is usually coupled with proteasomal degradation, forming a ubiquitin-proteasome system (UPS), which plays an essential role in the cellular processes for protein quality control. Protein quality control can occur in the cytoplasm, endoplasmic reticulum, or nucleus [2]. Within the nucleus, UPS is related to DNA replication, repair, maintaining genome integrity, and regulating gene expression [3,4].

The ubiquitination of proteins is a three-step enzymatic cascade that involves the ubiquitin-activating enzyme (E1), the ubiquitin-conjugating enzyme (E2), and the ubiquitin-protein ligase (E3)—this cascade results in the transfer of ubiquitin onto the substrate. E3 ubiquitin ligases, which directly recognize protein substrates, are the primary determinants of the specificity of protein ubiquitylation [5]. Classically, these E3s are classified into four protein families: RING (Really Interesting New Gene), HECT (homologous to E6AP C-terminus), U-box, and PHD-finger (Plant Homeo Domain). The RING family is also known as Cullin-RING Ligases (CRLs) due to its association with scaffold Cullin proteins. The RING family is the largest family of E3 and is responsible for the ubiquitination of ~20% of cellular proteins degraded through UPS. The Cullin protein family comprises eight members (CUL1 to CUL7 and PARC) [6]. The CRL containing CUL1 is also known as the SCF (Skp1–Cullin—F-box protein) complex. The SCF complex is composed of three invariable core components, namely the scaffolding protein Cullin 1 (CUL1), a RING-finger protein (RBX1) that recruits the E2, and the S-phase kinase-associated protein 1 (SKP1), an adaptor that bridges the core SCF complex with a variable F-box protein (FBP) and its corresponding protein target [7,8].

FBPs, with their characteristic F-box domain, are crucial components in the SCF E3. The term F-box was coined due to its presence in cyclin F [9]. The F-box motif, consisting of approximately 50 amino acids, serves as a platform for protein-protein interactions and is typically located in the N-terminal part of the FBP. The two most common types of FBPs are those with WD (Trp-Asp) repeats and leucine-rich repeats (LRRs). The FBP is connected to the core SCF by interacting with its F-box domain and the adaptor Skp1, while its WD repeats or LRRs bind substrates. The unique feature of FBPs is their ability to interchange in the SCF complex, acting as substrate-recognizing receptors and identifying specific substrates. This interchangeability dictates the substrate selectivity and specificity of the SCF [8,10].

Multiple life processes in fungi require a delicate equilibrium between protein synthesis and selective protein turnover via the UPS. The critical components of UPS are crucial for the fungal growth, development, and enzyme synthesis of various filamentous fungi such as *Aspergillus*, *Neurospora*, and *Trichoderma* [11]. During mycelial growth, 20% of the proteins in the nucleus of *Aspergillus nidulans* become ubiquitinated [12]. Ubiquitin ligase E3, such as the SCF complex, undergoes rapid FBP exchange to recognize different substrates to respond to physiological changes in the cell or adapt to external environments. Therefore, many cellular and developmental processes have been attributed to FBP function [10].

Originally, yeasts were the main subject of study for fungal FBPs. About 20 FBPs are present in *Saccharomyces cerevisiae* [13]. The number of FBPs is higher in filamentous fungi. *Aspergillus niger* and *A*. *nidulans*, for instance, have about 70 FBPs [14,15], while different *Fusarium* species have 60 ~ 95 FBPs [16]. FBPs play diverse roles in filamentous fungi. Several FBPs are involved in fungal growth, development, virulence, and extracellular enzyme secretion. *A*. *nidulans* Fbx15 and FBP GrrA (the homolog of Fbx50) are involved in fungal development [15,17]. The Fbx15 of the human pathogen *Aspergillus fumigatus* controls the nuclear location of the co-repressor SsnF, stress response, and virulence [18]. *A*. *nidulans* Fbx23 is required for fungal development and an appropriate secondary metabolism and is a regulator of CreA-mediated catabolite repression for hemicellulase (xylanase) production [15,19,20]. FBP Fwd1 of *Neurospora crassa* regulates the degradation of circadian clock protein FREQUENCY and proper circadian clock function [21]. Mutation of a gene (exo-1) encoding an FBP in *N*. *crassa* causes inducer-independent hypersecretion of amylases, invertase, and pectinases [22]. Excellent research on 74 FBPs in *A*. *nidulans* has shown that at least 45 FBPs are associated with more than 700 proteins [15]. However, the number of identified ubiquitin ligase-substrate pairs is limited. Most FBPs in eukaryotes remain “orphans”, i.e., their recognizing substrate has yet not been identified. Finding the substrates for these “orphans” remains a significant task for the field [23].

Many biological processes regulated by FBPs, such as Fbx23, depend on changes in the transcription levels of the biological process-related genes. It is interesting to wonder how FBPs—proteins that do not bind DNA directly—connect to the transcription of genes. Fungal growth, development, secondary metabolism, and extracellular enzyme secretion are strongly related to changes in the transcription levels of related genes, in which sequence-specific transcription factors play a crucial regulatory role [24,25]. The Cys2His2 (C2H2)-type transcription factor Ace1/SltA conserved in various filamentous fungi such as *Aspergillus*, *Penicillium*, *Trichoderma*, *Neurospora*, *Magnaporthe*, *Fusarium*, and *Beauveria*, plays a crucial role in regulating the expression of target genes. Ace1, a negative regulator for (hemi)cellulase gene transcription, was first discovered in *Trichoderma reesei* [26]. Deleting or disrupting the *ace1* gene in *T*. *reesei*, *Penicillium oxalicum*, and *Rasamsonia emersonii* increases the expression of the (hemi)cellulase genes [26–28]. The homolog of Ace1 is named SltA in *Aspergillus* sp. SltA is found to be involved in hyphal development, conidiation, sterigmatocystin biosynthesis, and virulence in different *Aspergillus* sp. [29–31]. These results highlight the variety of Ace1/SltA regulatory roles. However, it is yet unknown how Ace1/SltA is regulated into its active form.

*P*. *oxalicum* is a typical saprophytic fungus. It contains many transcription factors such as BrlA, which regulates fungal asexual development, CreA, XlnR, and Ace1, which regulate (hemi)cellulase gene transcription, conserved in various fungi such as *Aspergillus*, *Trichoderma*, and *Neurospora* [27,32]. These fungi share similar mechanisms for regulating asexual development and the transcription of (hemi)cellulase genes. For example, BrlA was reported as the key asexual developmental regulator in many *Aspergillus* sp. and *P*. *oxalicum* [33–35]. CreA/Cre1 has been found to repress cellulase gene expression by recruiting corepressor complex Tup1-Cyc8 in *P*. *oxalicum*, *T*. *reesei*, and *N*. *crassa* [36,37]. *P*. *oxalicum* can be employed as a model system to elucidate the regulatory mechanism of cellulolytic enzyme gene expression.

In this study, a ubiquitin ligase-substrate pair, SCF^Fbx23^-Ace1, is identified in *P*. *oxalicum*. The PoFbx23 recognizes and activates the transcription factor PoAce1 within the nucleus. PoAce1 is a ubiquitinated protein. In the absence of PoFbx23, PoAce1 accumulates in a dysfunctional form; only in the presence of PoFbx23 can PoAce1 bind to the promoter regions of the target gene in an active form. A model for the regulation of gene expression by PoFbx23-Ace1 is proposed.

## Results

### The SCF^Fbx23^ complex is among the interaction partners of transcription factor PoAce1

**Fig 1:**
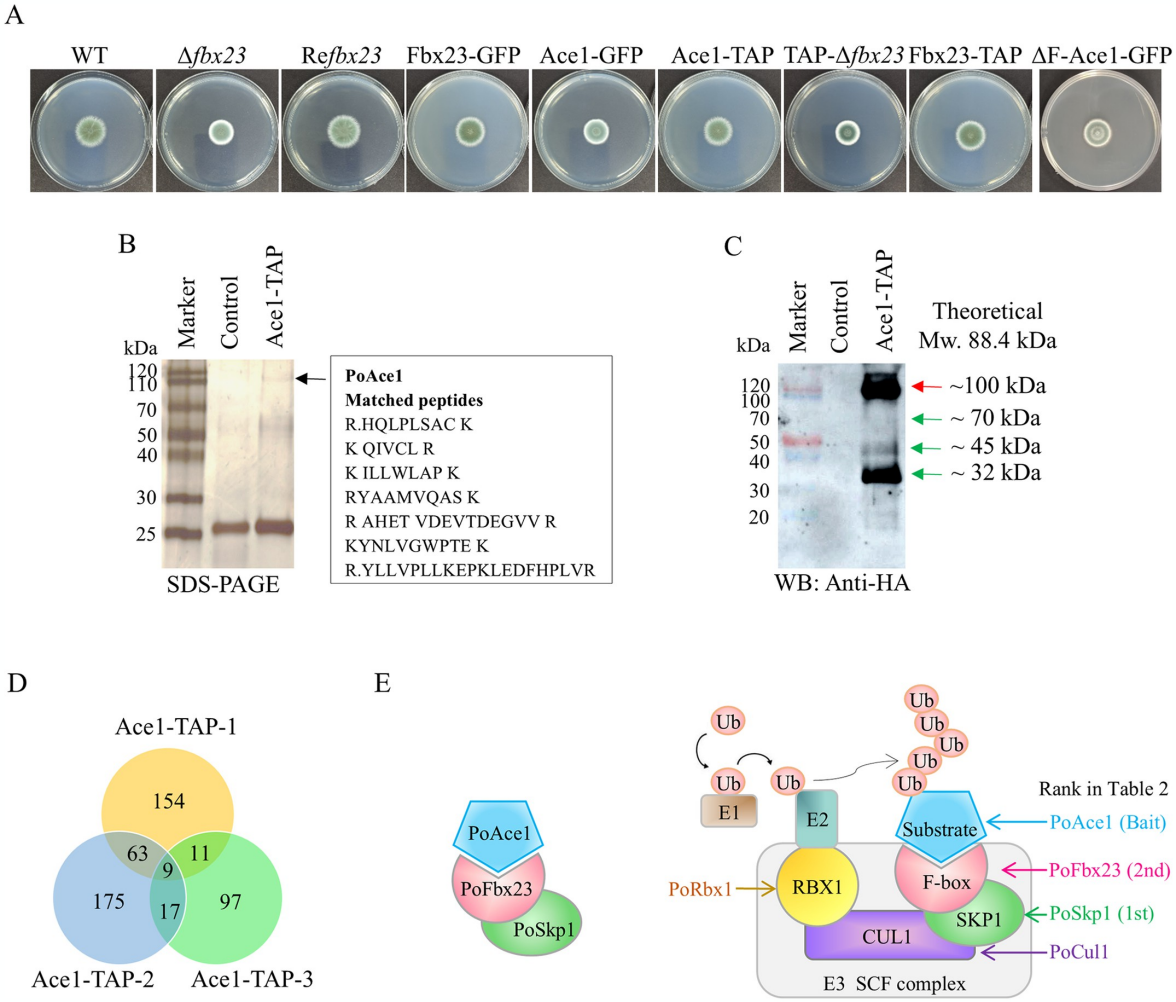
The results of TAP-MS using transcription factor PoAce1 as bait. The Ace1-TAP strain was generated by fusing the TAP tag (HA-FLAG) at the C-terminus of the PoAce1 (PDE_01988, Uniprot Entry S8AYJ0). The WT strain 114–2 containing native PoAce1 was used as a control. The biological triplicates were used for the TAP-MS experiment. **(A)** Colonies of *P*. *oxalicum* wild-type (WT) strain and strains constructed in this study. 1 μl conidia suspension (10^7^ conidia ml^−1^) of various strains was point-inoculated onto Vogel’s minimal medium (VMM) plus 2% glucose (VMMG) agar and then cultivated at 30°C for three days. **(B)** Silver staining after SDS-PAGE of Ace1-TAP eluate. The arrow represents the protein PoAce1. **(C)** Western blot of Ace1-TAP eluate using anti-HA antibody. **(D)** Intersection proteins among Ace1-TAP biological triplicates (Ace1-TAP-1/2/3) (The detailed information of proteins is shown in Table 2, S1 Spreadsheet and S1 Data). **(E)** The models of heterodimer Fbx23-Skp1 **(left)** and ubiquitin ligase complex SCF^Fbx23^ **(right)** according to the TAP-MS results. The bait PoAce1 is the substrate. The PoFbx23 (ranked 2nd in Table 2) is the F-box-containing protein that interacts with the substrate PoAce1 and determines substrate specificity. The PoSkp1 (ranked 1st in Table 2) is an adapter protein of the SCF complex. PoCul1 (ranked 10th in S1 Spreadsheet, sheet1, row 13, yellow background) is the scaffolding protein interacting with the SKP1-F-box complex and RBX1. PoCul1 (ranked 73rd in S1 Spreadsheet, sheet1, row 75, yellow background) is the RING-finger protein that recruits the E2 ubiquitin-conjugating enzyme.

All strains used in this study are listed in Table 1. Colonies of *P*. *oxalicum* wild-type (WT) strain and strains constructed in this study are shown in Fig 1A.

**Table 1:** Strains used in this study.

Strains	Genotype	Descriptions	References
*P*. *oxalicum*			
114–2	Wild type (WT)	Wild type CGMCC5302*	[32]
Δ*ace1*	Δace*1*::*hph*	Deletion *ace1* (*ace1*) gene in the WT	[27]
Ace1-TAP	*ace1*::TAP::*hph*	Ace1 fused with HA-FLAG tag in C-terminus of the protein	This study
Ace1-GFP	*ace1*::GFP::*hph*	Ace1 fused with GFP in C-terminus of the protein	This study
Fbx23-GFP	*fbx23*::GFP::*hph*	Fbx23 fused with HA-FLAG tag in C-terminus of the protein	This study
Fbx23-TAP	*fbx23*::TAP::*hph*	Fbx23 fused with GFP in C-terminus of the protein	This study
Ace1-YFP-Fbx23	^#^*P_PDE_01335_*::*Ace1*::NYFP::Ter1, *ptrA*; P*gpdA*::*fbx23*:: CYFP::Ter2, *hph*	The co-expression strain of Ace1 fused with N-YFP sequence and Fbx23 fused with C-YFP sequence	This study
Δ*fbx23*	Δ*fbx23*::*hph*	Deletion *fbx23* gene in the WT	This study
Re*fbx23*	Δ*fbx23*::*hph fbx23*::*ptrA*	Recomplement with *fbx23* gene on the basis of Δ*fbx23*	This study
ΔF-Ace1-GFP	Δ*fbx23*::*hph ace1*::GFP::*ptrA*	The Ace1 protein fused the GFP in the Δ*fbx23* background	This study
TAP-Δ*fbx23*	*Ace1*::TAP::*hph* Δ*fbx23*::*ptrA*	Deletion *fbx23* gene on the basis of Ace1-TAP	This study
*S*. *cerevisiae*			
Y2H Gold-BD-Fbx23	Y2H Gold-pGBKT7-fbx23	The coding domain sequence (CDS) of *fbx23* gene was coloned into plasmid pGBKT7 and transformed into strain Y2H	This study
Y187-AD-Ace1 (1–396 aa)	Y187-pGADT7-Ace1 (1–396 aa)	The CDS (1–396 aa) of *ace1* gene was coloned into plasmid pGADT7 and transformed into strain Y187	This study
Y187-AD-Ace1 (484–816 aa)	Y187-pGADT7-Ace1 (484-816aa)	The CDS (484–816 aa) of *ace1* gene was coloned into plasmid pGADT7 and transformed into strain Y187	This study
Y187-AD-T	Y187-pGADT7-T	The strain Y187 containing plasmid pGADT7 which carries Gal4 AD fused with SV40 large T-antigen	The lab
Y2H Gold-BD-p53	Y2H Gold-pGBKT7-p53	Positive control as BD fused p53	The lab
Y2H Gold-BD-lam	Y2H Gold-pGBKT7-lam	Negative control as BD fused Lam	The lab

*: CGMCC, China General Microbiological Culture Collection Center.

#: P*_PDE_01335_*, the PDE_01335 encodes a phosphoglycerate mutase family protein. The expression of gene PDE_01335 is high and can be used as a constitutive promoter for gene expression [38].

Tandem affinity purification coupled with mass spectrometry (TAP-MS) is a valuable technique for identifying interacting partners of the protein of interest in the studies of yeast, *Aspergillus*, *Trichoderma*, and *Penicillium* [36,39,40]. The eluate of the Ace1-TAP strain displayed a specific band at about 100 kDa in comparison to the control strain, as shown by the SDS-PAGE result (Fig 1B, dark arrow, PoAce1’s theoretical molecular weight with the TAP tag added is 88.4 kDa). The specific band was then cut out of the gel and was confirmed to be the PoAce1 protein through MS/MS assay (S1 Document). This finding was supported by Western blot using anti-HA antibody (Fig 1C, red arrow). In addition to the signal at about 100 kDa, the Western blot result revealed several low-molecular-weight signals at about 70-, 45- kDa, and 32-kDa (Fig 1C, green arrows).

The proteins in the third section of the eluent were analyzed using LC-MS/MS to identify the bait and interacting proteins. The PepCount identified in biological triplicates (Ace1-TAP-1, Ace1-TAP-2, and Ace1-TAP-3) through TAP-MS were listed in S1 Spreadsheet (Sheet 2 ~ Sheet 4). The proteins observed in any three Ace1-TAP samples but not in any of the controls were listed in S1 Spreadsheet (Sheet 1). The proteins were ranked by exponentially modified protein abundance index (emPAI), which was used to estimate protein abundance in the sample [41]. 237, 264, and 134 proteins were identified in the samples Ace1-TAP-1, Ace1-TAP-2, and Ace1-TAP-3, respectively. Nine proteins were observed in all three Ace1-TAP samples but not in any of the controls (Fig 1D). They were considered reliable proteins that have putative interaction with the PoAce1 and were listed in Table 2.

**Table 2:** The top proteins interacting with PoAce1 identified through TAP-MS.

Rank	Gene Locus	Uniprot Entry	emPAI^a^	*oxalicum* 114–2	*cerevisiae* S288C^b^			Predicted function
				Protein	Homolog	Identity %	Location	
Bait	PDE_01988	S8AYJ0	12.15	PoAce1	-	-	-	Cys2-His2 zinc finger transcription factor
1st	PDE_02249	S7ZF79	9.00	PoSkp1	SKP1	50	Nucleus/ Cytoplasm	Component of the SCF E3 ubiquitin ligase complexes
2nd	PDE_08467	S7ZS07	3.58	PoFbx23	Pof11^c^	45	Nucleus/ Cytoplasm	Component of the SCF E3 ubiquitin ligase complexes
3rd	PDE_09763	S7ZWH9	0.44	-	-	-	-	Uncharacterized protein
4th	PDE_05651	S7ZK70	0.22	PoPyc2	PYC2	71	Cytoplasm	Pyruvate carboxylase
5th	PDE_01581	S8ALA0	0.19	PoAsh1	Set2	36	Nucleus	Histone-lysine N-methyltransferase
6th	PDE_05666	S7ZK88	0.17	PoMub1	MUB1	52	Nucleus/ Cytoplasm	MYND domain-containing protein; component of the Mub1/Ubr2 ubiquitin ligase complex
7th	PDE_00208	S7Z423	0.13	-	-	-	-	Uncharacterized protein
8th	PDE_05998	S7ZL42	0.13	-	-	-	-	Uncharacterized protein
……	……		……	……	……	……	……	……
&	PDE_02289	S7ZFE3	0.77	PoRbx1	RBX1/ HRT1	64	Nucleus/ Cytoplasm	Component of the SCF E3 ubiquitin ligase complexes
&	PDE_00128	S7Z946	0.07	PoCul1	CDC53/ Cullin-1	31	Nucleus/ Cytoplasm	Component of the SCF E3 ubiquitin ligase complexes

^a^: emPAI is the Exponentially Modified Protein Abundance Index of three samples. Every Peptide count of each sample is listed in the S1 Spreadsheet (Sheet 2–4).

^b^: Data from *S*. *cerevisiae* Genome Database (www.yeastgenome.org).

^c^: Data from *Schizosaccharomyces pombe* Genome Database (www.pombase.org).

&: Only observed in two of the three biological samples.

-: No significant similarity found.

As expected, the bait PoAce1 has the highest emPAI. Among the other eight proteins, the protein with the highest emPAI is PDE_02249 (Uniprot Entry S7ZF79). We name PDE_02249 as PoSkp1 because its homolog in *S*. *cerevisiae* is protein SKP1, which is an invariable, core component of the SCF E3 ubiquitin ligase complex that functions as the adaptor protein responsible for binding CUL1 and recruiting various F-box proteins for SCF complex formation [8].

The protein with the second-highest emPAI is PDE_08467 (Uniprot Entry S7ZS07), an F-box/WD repeat protein which is always a variable component of SCF complex that binds to SKP1 through the F-box domain [9]. The homologous protein of PDE_08467 is not detected in *S*. *cerevisiae*. However, it was identified in *Schizosaccharomyces pombe* to be the F-box/WD repeat protein Pof11 (Genbank: NP_594559.1) and in *A*. *nidulans* to be the F-box containing protein AnFbx23 (ANIA_05593, Genbank: XP_663197.1). We name PDE_08467 as PoFbx23. Like AnFbx23, PoFbx23 possesses an F-box domain and seven copies of WD repeats (S1 Fig.), which act as a platform for protein-protein or protein-DNA interaction [42].

The proteins ranked third (PDE_09763), seventh (PDE_00208), and eighth (PDE_05998) are uncharacterized proteins. The proteins ranked 4th (PDE_05651) and 6th (PDE_05666) are named PoPyc2 and PoMub1 because their homologs in *S*. *cerevisiae* are PYC2 and MUB1, respectively. The protein ranked 5th (PDE_01581) shares similarities (36%) with Set2 in *S*. *cerevisiae*. As *P*. *oxalicum* contains a protein (PDE_02255, Uniprot Entry: S8AZ99) that shares a higher identity (45%) with the *S*. *cerevisiae* Set2 and has been named PoSet2 [43], the PDE_01581 is named PoAsh1 because its homolog in *Homo sapiens* and *Mus musculus* is histone methyltransferase Ash1. We also observe PDE_02289, a RING-type domain-containing protein, and PDE_00128, a Cullin family domain-containing protein (Table 2). PDE_02289 and PDE_00128 are present in two but not three biological replicates (S1 Spreadsheet, sheet 1, row 13 and row 75). The PDE_02289 is named PoRbx1 because its homolog is protein RBX1, a subunit of the SCF complex that tethers the E2 and Cullin subunits [8]. The PDE_00128 is named PoCul1 because its homolog is the protein Cullin-1, which functions as a molecular scaffold that simultaneously interacts with the SKP1-F-box complex and with RBX1 or RBX2 [11].

The discoveries of PoSkp1, PoFbx23, PoRbx1, and PoCul1 suggest there exist four components of the SCF^Fbx23^ E3 ubiquitin ligase complex in the Ace1-TAP eluent. However, it is noteworthy that the top three proteins, bait (PoAce1), PoSkp1, and PoFbx23, which were observed in all three biological samples, had high emPAI values (12.15, 9.00, and 3.58, respectively). In contrast, the other two subunits (PoRbx1 and PoCul1) of the SCF complex, which only appeared in two of the three biological samples, had low emPAI values (0.77 and 0.07, respectively) (Table 2). As previous studies have shown that substrate adaptors do not always associate with Cul1-Rbx1 heterodimers [44]; assembly of F-box proteins into SCF complexes ranges from 0–70% (median of ~19%) [45], we speculate that the majority of PoFbx23 binds PoAce1 by forming a heterodimer PoFbx23-Skp1 (Fig 1E, left), while a small portion of PoFbx23 assembles with the other three components to form the SCF^Fbx23^ complex. In the SCF^Fbx23^ complex, PoCul1 acts as a molecular scaffold, PoRbx1 binds to ubiquitin-loaded E2, PoSkp1 serves as an adaptor, and PoFbx23 is a substrate receptor protein determining the substrate specificity. The PoFbx23 recognizes PoAce1 as the substrate (Fig 1E, right). In addition, the discovery of PoMub1 is also noteworthy. PoMub1, as a component of the Ubr2/Mub1 ubiquitin ligase complex, implies that PoAce1 may not be confined to just one ubiquitin ligase system.

### PoAce1 and PoFbx23 are localized in the nucleus and interact directly with each other there

Transcription factors can be localized within the nucleus or shuttle from the cytoplasm into it in response to different signals [46]. Moreover, SCF E3 can also be localized in the nucleus and cytoplasm [47,48]. Even for a particular type of FBP, such as FBXW7, its different isoforms can still be localized to the nucleus, cytoplasm, and nucleolus [49]. In light of this, even though TAP-MS results indicate that PoFbx23 might recognize and bind PoAce1, the precise location of this recognition remains unknown. In addition, the putative collaborators identified by TAP-MS contain not only the true interactors of the bait protein but also the proteins that indirectly interact with the bait protein mediated by other proteins when multiple proteins form complexes. Therefore, we first identified the subcellular localization of PoAce1 and PoFbx23 and then determined whether there is a direct interaction between PoAce1 and PoFbx23 and the subcellular location where the interaction occurs using the methods of yeast two-hybrid (Y2H) and bimolecular fluorescence complementation (BiFC).

**Fig 2:**
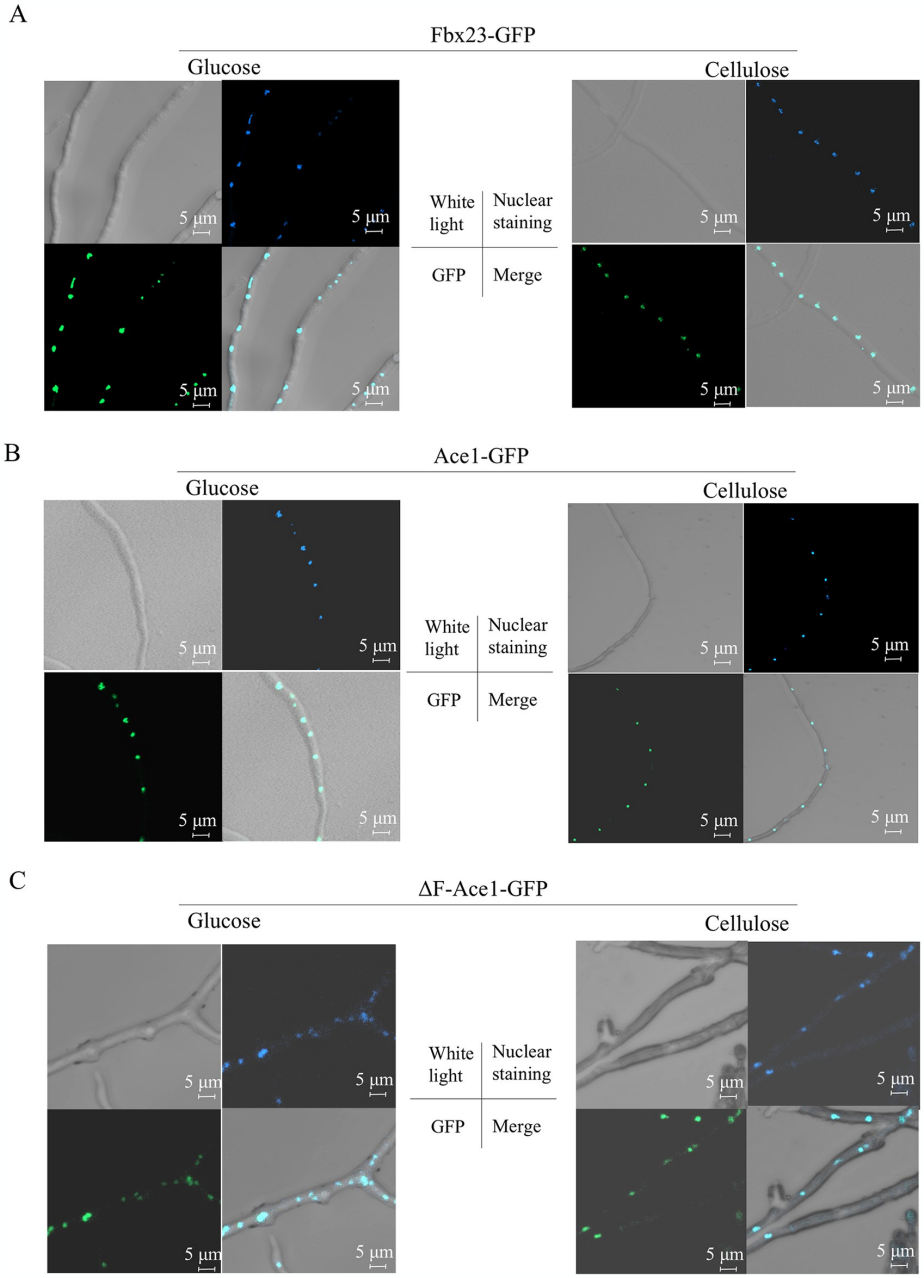
Subcellular localization of PoFbx23A and PoAce1. **(A)** Subcellular localization of PoFbx23 on VMMG (left) and VMMC (right), respectively. **(B)** Subcellular localization of PoAce1 on VMMG (left) and VMMC (right), respectively. **(C)** Subcellular localization of PoAce1 in the Δ*fbx23* background on VMMG (left) and VMMC (right), respectively. The image is divided into four parts: upper left, white light; upper right, Hoechst 33342 was used to stain nuclei in blue; bottom left, green fluorescence; bottom right, merged image of green fluorescence and nuclear staining.

The strains Ace1-GFP and Fbx23-GFP were constructed. Due to the various signals that can trigger different subcellular localization of transcription factors and FBPs, we observed the subcellular localization of PoAce1 and PoFbx23 on Vogel’s minimal medium (VMM) plus 2% glucose (VMMG) and VMMC (plus 2% cellulose), respectively. The VMM is a universal and convenient minimal medium for studying various fungi such as *Neurospora*, *Aspergillus*, and *Penicillium* in the lab [27,50,51]. The glucose in VMMG inhibits cellulolytic gene transcription due to carbon catabolite repression (CCR) [52]. The VMMC is a suitable medium for studying regulatory proteins for cellulolytic gene transcription due to the inducer cellulose. An overlap of green fluorescence (Fig 2 A & 2B, bottom left) and nuclear staining (Fig 2A & 2B, top right) was observed on the merged image (Fig 2A & 2B, bottom right), regardless of whether the strains Fbx23-GFP and Ace1-GFP were cultured on VMMG or VMMC (Fig 2A & 2B). To observe whether the subcellular localization of PoAce1 is affected by PoFbx23, the mutant ΔF-Ace1-GFP (the Ace1 protein fused the GFP in the Δ*fbx23* background) was constructed. PoAce1 was localized in the nucleus in the absence of PoFbx23, whether under glucose or cellulose signal (Fig 2C). The results indicate that PoFbx23 and PoAce1 are both localized in the nucleus, whether under glucose or cellulose signal; the localization of PoAce1 is not affected by PoFbx23.

**Fig 3:**
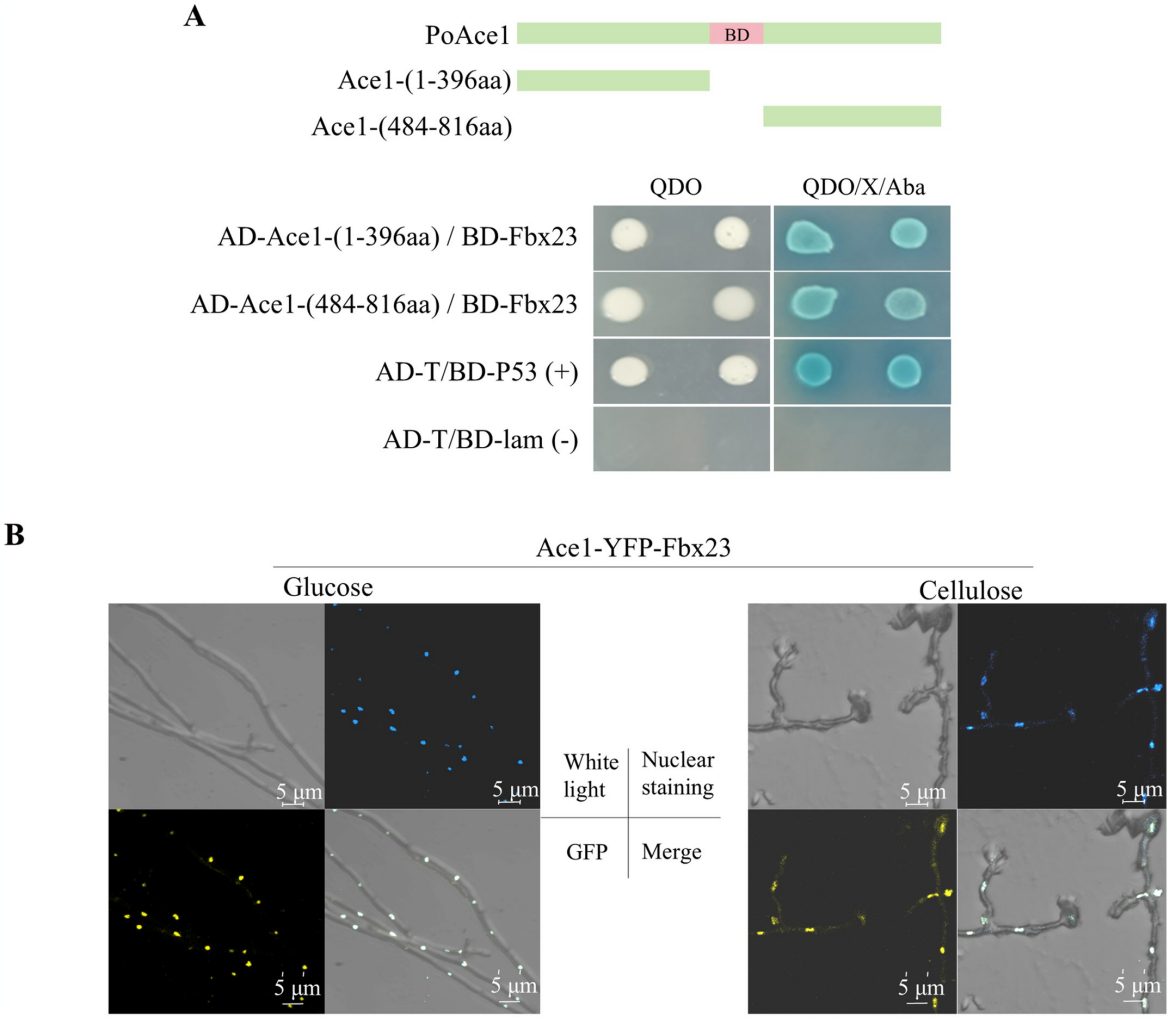
The interaction between PoFbx23A and PoAce1. **(A)** Yeast two-hybrid strategy and hybridization results of Y2H Gold-BD-PoFbx23 and Y187-AD-PoAce1 strains on QDO (quadruple-dropout, SD-Ade/-His/-Leu/-Trp) and QDO/x-α-gal/Aba (QDO supplemented with X-α-gal and aureobasidin A). DNA-binding domain (397–483) was removed from PoAce1 as the intact protein showed autoactivation. **(B)** Microscopy of BiFC strain Ace1-YFP-Fbx23 on VMMG (left) and VMMC (right), respectively. The image is divided into four parts: upper left, white light; upper right, Hoechst 33342 was used to stain nuclei in blue; bottom left, yellow fluorescence; bottom right, merged image of yellow fluorescence and nuclear staining.

Y2H results showed two fragments of PoAce1 had evident interaction with PoFbx23, suggesting both fragments may contain regions that interact with PoFbx23 (Fig 3A). The Ace1-YFP-Fbx23 strain for BiFC was constructed. An overlap of yellow fluorescence (Fig 3B, bottom left) and nuclear staining (Fig 2, top right) was observed on the merged image (Fig 3B, bottom right), regardless of whether the strain Ace1-YFP-Fbx23 was cultured on VMMG or VMMC (Fig 3B). These results strengthen the reliability and certainty of the direct interaction between the PoFbx23 and PoAce1 and demonstrate that the interaction occurs inside the nucleus regardless of the glucose or cellulose signal.

### PoFbx23 absence under the glucose signal resulted in decreased transcription of the *brlA* gene and spore pigmentation genes, along with impaired conidiation

TAP-MS, Y2H, and BiFC results demonstrate that the PoFbx23 recognizes transcription factor PoAce1 as the substrate in the nucleus. Given that SltA (the homolog of Ace1) is known to be crucial in fungal growth, development, and secondary metabolite biosynthesis in various fungi [29,30,31], it is reasonable to assume that PoFbx23 could indirectly influence these biological processes. However, the nature of this influence, whether positive (activating) or negative (repressing), remains uncertain. Therefore, we constructed the Po*fbx23* gene deletion (Δ*fbx23*) mutant and conducted phenotype, RT-qPCR, and transcriptome analyses to explore this assumption.

**Fig 4:**
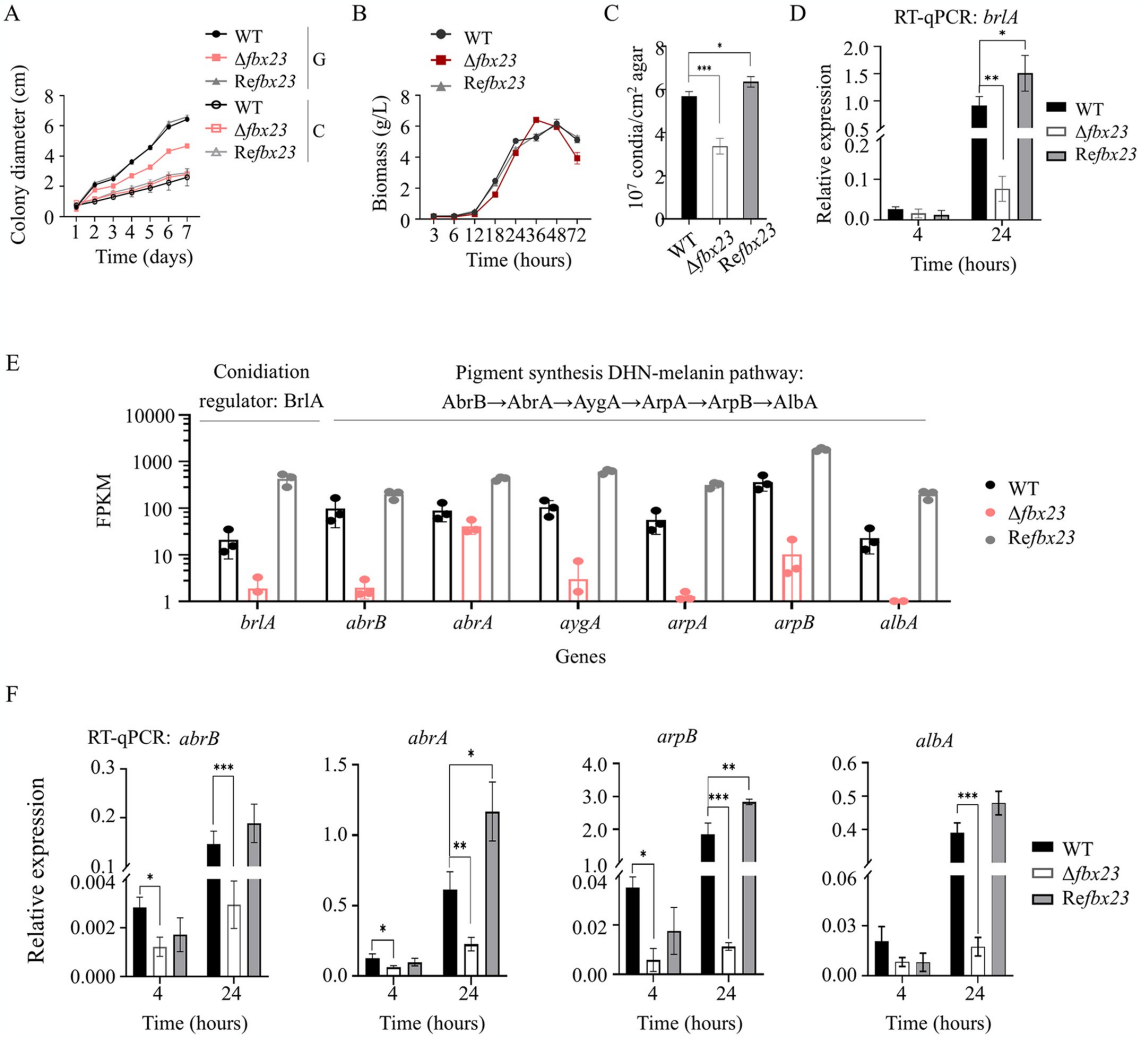
Effects of PoFbx23 protein on fungal growth, conidiation, and the transcription of genes involved in conidiation and spore pigment synthesis under glucose signal. (A) Colony diameter determination. 1 μl conidia suspension (10^7^ conidia ml-^1^) of various strains was point-inoculated onto the VMMG or VMMC agar and cultivated at 30°C. G, VMMG. C, VMMC. All P values of radial growth of the Δ*fbx23* strain compared to the WT when they were cultivated in VMMG are < 0.05. (B) The biomass measurement of different strains. **(C)** Conidia quantification. The WT, Δ*fbx23*, and Re*fbx23* strains were incubated on VMMG agar at 30°C for 5 days. Then, 5-mm diameter colony agar plugs were harvested with the physiological salt (0.2% w/v Tween 80 and 0.8% w/v NaCl). Conidia quantification was determined based on a direct count by using a hemocytometer. **(D)** Transcription level of the *brlA* gene assayed by qRT-PCR. **(E)** The expression of the *brlA* gene and six genes *abrB*, *abrA*, *aygA*, *arpA*, *arpB*, and *albA* involved in the DHN-melanin pathway of pigment synthesis revealed by transcriptome data after the strains were incubated in VMMG liquid. The differential expression folds change and the Q-value of each gene between the mutant and the WT are shown in S2 Spreadsheet (Shee1). All Q-values are < 0.05. The copy number of unambiguous tags mapping to genes was normalized to fragments per kilobase transcriptome per million mapped reads (FPKM). **(F)** The transcription level of four genes, *abrB*, *abrA*, *arpB*, and *albA* assayed by qRT-PCR. The values were normalized to the *actin* gene levels considered 1. *p < 0.05, **p < 0.01, ***p < 0.001.

Cultivated on VMMG agar, the Δ*fbx23* mutant showed a reduced rate of colony expansion than that of the WT (Fig 4A). The hyphal elongation speed of the Δ*fbx23* colony is about 0.69 cm per day, a 21% decrease from the WT of about 0.90 cm per day. However, the growth patterns of the strains were almost identical when they were cultivated in VMMG liquid, as shown by the biomass measurement (Fig 4B). When strains WT, Δ*fbx23*, and Re*fbx23* were grown on VMMC agar, there was no significant difference in their radial growth. However, the colony diameters of these three strains were significantly smaller on VMMC than on VMMG. For example, on the 7^th^ day of incubation, the colony diameters of WT and Δ*fbx23* on VMMC agar were only 40.6% and 59.6% of those on VMMG agar, respectively (Fig 4A).

The conidiation in Δ*fbx23* was reduced to approximately 63.2% of that in the WT (Fig 4C). As transcription factor BrlA is a master regulator for asexual development and its regulatory role cannot be bypassed during conidiation of *Penicillium* and *Aspergillus* [34,35], *brlA* expression was assayed using RT-qPCR after the strains were cultivated in VMMG liquid. The deletion of Po*fbx23* gene downregulated the expression of the *brlA* gene significantly (Fig 4D). Transcriptome data from Δ*fbx23* cultured in the same medium also supported the decrease in expression of the *brlA* gene; the transcription level of the *brlA* gene in the Δ*fbx23* reduce to only 9.1% of that in the WT (Fig 4E).

Interestingly, in addition to the downregulation of the *brlA* gene, downregulated expression of genes related to spore pigment synthesis was observed when we investigated the transcriptome data. Melanin, the critical spore wall pigment, provides the characteristic dark green pigment for many fungal spores. Melanin is synthesized through the dihydroxynaphthalene (DHN)-melanin pathway that includes six proteins (Abr2→Abr1→Ayg1→Arp1→Arp2→Alb1) in *A*. *fumigatus* [53]. The homologs of the above six proteins in *P*. *oxaliucm* are laccase gene AbrB/yA (PDE_09492, Genbank No. EPS34528.1), pigment biosynthesis protein brown 1 AbrA (PDE_09453, Genbank No. EPS 34489.1), pigment biosynthesis protein AygA (PDE_09452, Genbank No. EPS34488.1), tetrahydroxy-naphthalene reductase ArpA (PDE_04496, Genbank No. EPS29546.1) and ArpB (PDE_04495, Genbank No. EPS29545.1), and polyketide synthase gene AlbA/wA (PDE_09491, Genbank No. EPS34527.1), respectively. The transcription of all six pigment biosynthesis-related genes was significantly downregulated in the Δ*fbx23*. The transcription level of *abrB*, *abrA*, *aygA*, *arpA*, *arpB*, and *albA* gene in the Δ*fbx23* reduced to 2.0%, 46.3%, 2.8%, 2.3%, 2.8%, and 4.1% of that in the WT, respectively (Fig 4E, right). The RT-qPCR assay of the first two genes (*abrB* and *abrA*) and the last two genes (*arpB* and *albA*) in the DHN-melanin pathway supported the transcriptome results; the transcription level of *abrB*, *abrA*, *arpB*, and *albA* gene in the Δ*fbx23* reduce to 2.0%, 36.8%, 0.6%, and 4.5% of that in the WT at cultivation time of 24 h (Fig 4F and S2 Data).

Deleting Po*fbx23* gene also affected the expression of secondary metabolic gene clusters in addition to fungal growth and asexual development. There are 28 secondary metabolism gene clusters predicted in the *P*. *oxalicum* genome (S1 Table) [32]. Among the 28 secondary metabolism gene clusters, four secondary metabolism gene clusters, which correspond to clusters 1, 2, 26, and 28, respectively (S1 Table, yellow background), were downregulated in the Δ*fbx23* mutant (S2A~D Fig). Among four secondary metabolism gene clusters, the two clusters presented in S2B Fig (Cluster 2) and S2C Fig (Cluster 26) have cluster synteny with *Penicillium chrysogenum*. Cluster 2 is involved in the biosynthesis and secretion of the mycotoxins roquefortine C and meleagrin [54]. Interestingly, all four secondary metabolism gene clusters, silenced in the Δ*fbx23*, were also silenced in the previously reported *brlA* deletion mutant [35]. This finding implies that the four secondary metabolic gene clusters are indirectly downregulated due to the Po*fbx23* deletion-induced downregulation of *brlA* expression.

In conclusion, the involvement of PoFbx23 in hyphal development, conidiation, and secondary metabolite gene expression is comparable to the identification of SltA/Ace1 in these same processes in various fungi [29–31]. In addition, the phenotype of the Po*ace1* gene deletion mutant (Δ*ace1*) of *P*. *oxalicum* was observed (S3 Fig). The Δ*ace1* mutant showed a slower hyphal elongation speed than the WT (S3A&S3B Fig). It also showed a significant decrease in conidiation and the transcription levels of the *brlA* and *abrB* genes (S3C~E Fig). The results further suggested a correlation between PoFbx23 and PoAce1 in regulating hyphal development and conidation.

### PoFbx23 absence under the glucose signal does not affect Poace1 gene transcription but results in an accumulation of PoAce1 protein

**Fig 5:**
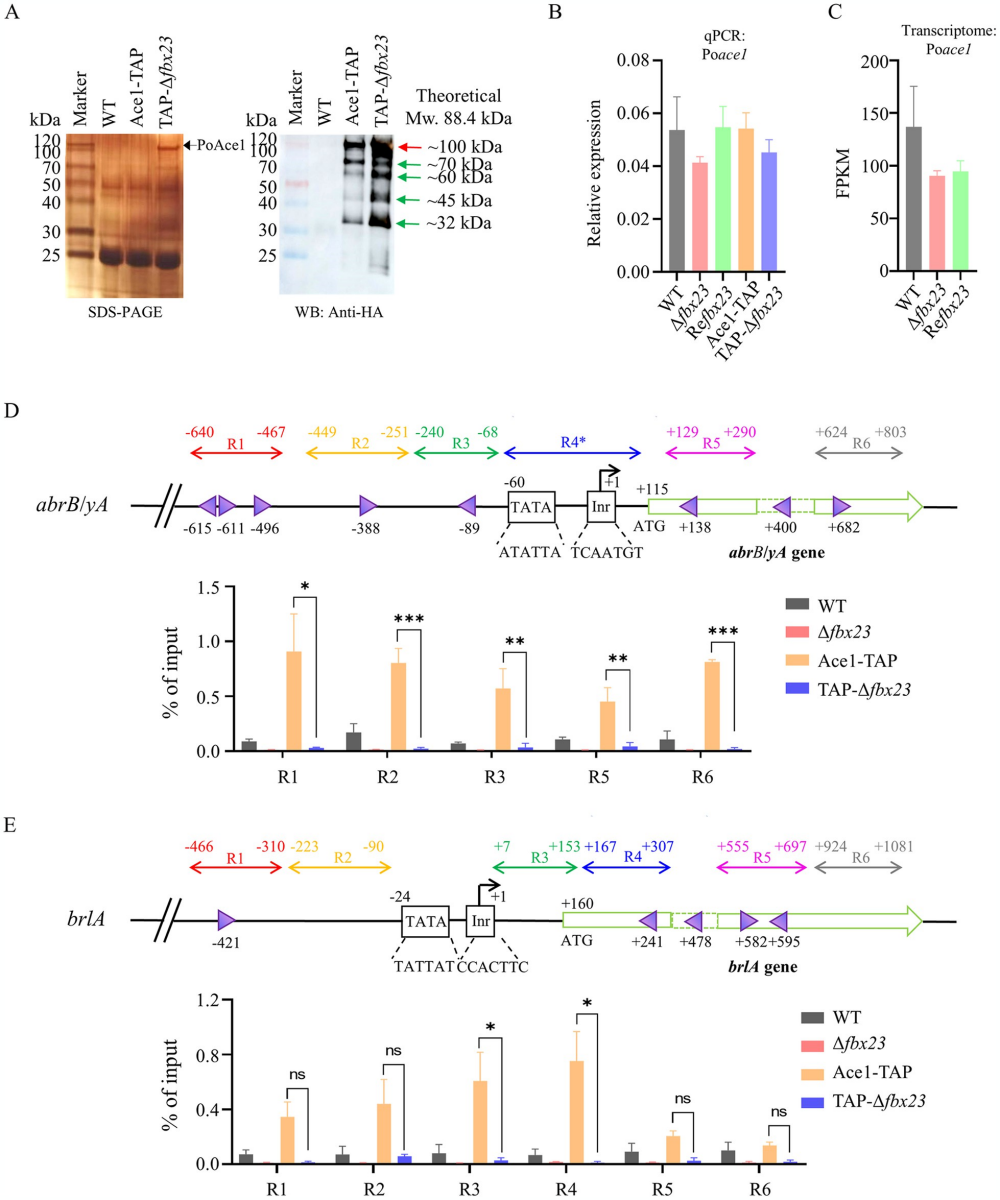
The effect of PoFbx23 on the protein abundance, transcription level, and DNA binding ability of PoAce1 under glucose signal. The strains were grown in VMMG. **(A)** The results of TAP using PoAce1 as bait in the Ace1-TAP and TAP-Δ*fbx23* strains. **Left**, silver staining after SDS-PAGE of Ace1-TAP eluate (The dark arrow represents the PoAce1 protein (about 100 kDa on the gel, theoretical MW: 88.4 kDa). **Right**, the Western blot of Ace1-TAP eluate using an anti-HA antibody. The arrow represents the PoAce1 protein. **(B)** The transcription level of the Po*ace1* gene in the WT, Δ*fbx23*, Re*fbx23*, Ace1-TAP, and TAP-Δ*fbx23* strains, assayed by RT-qPCR. The values were normalized to the *actin* gene levels considered 1. **(C)** The transcription level of Po*ace1* gene in the WT, Δ*fbx23*, Re*fbx23*, and revealed by transcriptome data. The copy number of unambiguous tags mapping to genes was normalized to FPKM. **(D)** The PoAce1 protein enrichment in the specific regions of the *abrB* gene assayed by ChIP-qPCR. **Top subgraph**, Strategy of ChIP-qPCR. The transcription start point (TSP) is designated as +1. The initiator (Inr) and TATA box are illustrated. Six specific regions (R1 to R6) of each gene are designed for qPCR assay (as shown at the top of the subgraph). Region 1 (R1), region 2 (R2), and region 3 (R3) are located upstream of the TSP. Region 4 (R4) covers the TATA-box and the initiator (Inr) (R4*, no appropriate primers for qPCR could be designed within this region). Region 5 (R5) is located in the 5′ region of the CDS of the *abrB* gene. Region 6 (R6) is located in the middle of the CDS. **Bottom subgraph**, the results of ChIP-qPCR for *abrB* gene. (E) The PoAce1 protein enrichment in the specific regions of the *brlA* gene assayed by ChIP-qPCR. Top subgraph, Strategy of ChIP-qPCR. The TATA box and Inr were not covered by the region design, because no appropriate primers for qPCR could be designed within this region. Bottom subgraph, the results of ChIP-qPCR for *brlA* gene. Purple triangles indicate the putative DNA-binding sites of PoAce1. The orientation of the triangle represented the orientation of the binding motif. The relative enrichment of IP DNA was calculated by % of input. All values are means from measurements of biological triplicates. The error bars indicate standard deviations *p < 0.05, **p < 0.01, ***p < 0.001.

Then, we investigated how PoFbx23 absence affected PoAce1 under glucose signal (Fig 5). The Po*fbx23* gene was deleted in the Ace1-TAP strain to construct the TAP-Δ*fbx23* mutant. Using the TAP method, PoAce1 was obtained from mycelial cells of the same biomass of WT, Ace1-TAP, and TAP-Δ*fbx23* strains after they were cultured in VMMG liquid. When PoFbx23 is present in the Ace1-TAP strain, PoAce1 exists as a full-length version (about 100 kDa, theoretical molecular weight is 88.4 kDa), and several low-molecular-weight degraded versions (about 70-, 60-, 45-, and 32-kDa). The abundance of intracellular PoAce1 was significantly increased in the TAP-Δ*fbx23* strain. While the band of PoAce1 was nearly invisible on the SDS-PAGE of the eluate of the Ace1-TAP strain, they were visible at ~100 kDa on the TAP eluate of the TAP-Δ*fbx23* strain (Fig 5A, left). The Western Blot results also demonstrated a stronger PoAce1 signal at about 100-, 70-, 60-, 45-, and 32-kDa of the TAP-Δ*fbx23* strain than that of Ace1-TAP (Fig 5A, right). The findings demonstrate that PoAce1 protein is more stable and accumulates in the cell in the absence of PoFbx23.

Initially, we hypothesized that the increase in PoAce1 protein abundance was due to the increased transcription of its genes. Therefore, RT-qPCR was used to determine the transcription levels of the Po*ace1* gene in WT, Δ*fbx23*, Re*fbx23*, Ace1-TAP, and TAP-Δ*fbx23* grown in VMMG liquid. Neither the Δ*fbx23* mutant nor the TAP-Δ*fbx23* mutant showed a change in the transcript levels of the Po*ace1* gene compared to their respective parent strains, the WT and Ace1-TAP (Fig 5B). Additionally, the transcriptome data of the WT and Δ*fbx23* mutant grown in VMMG also showed that the transcription levels of Po*ace1* gene were not significantly different between the WT and the Δ*fbx23* mutant (Fig 5C). The results demonstrate that the deletion of Po*fbx23* had no effect on Po*ace1* gene transcription but led to the accumulation of PoAce1 protein under glucose signal. Given the biological function of the SCF complex, it is reasonable to assume that PoAce1 protein accumulates in the cell due to PoFbx23’s absence which prevents PoAce1 protein from being degraded via the nuclear UPS pathway.

### Accumulated PoAce1 no longer binds pigmentation-related gene *abr2*

The Po*fbx23* deletion strain exhibited an accumulation of PoAce1 protein, accompanying downregulated expression of the *brlA* gene, pigmentation-related genes, and impaired conidiation. This finding contradicts earlier research that suggested a positive correlation between the transcription factor Ace1 and *brlA* expression and conidiation [29,30]. The activating activity of PoAce1 seems lost due to Po*fbx23* deletion. According to previous research on SltA/Ace1 in *Aspergillus* sp. and *Trichoderma* sp., SltA/Ace1 regulates the expression of target genes by directly binding to the conserved 5′-AGGCA-3′ motif in their promoter regions [26,55]. Our analysis of the promoter region of the pigmentation-related gene *abr2*, which encodes the first enzyme in the DHN-melanin pathway, revealed a significantly higher frequency of motif occurrence (5 sites of 5′-AGGCA-3′) than the theoretical frequency (2 sites) (Fig 5D, upper). This finding suggests that PoAce1 might bind directly to the *abrB* gene. To confirm this, we used Chromatin immunoprecipitation (ChIP)-qPCR to detect the degree of enrichment of PoAce1 in the promoter of the *abrB* gene in the WT, Δ*fbx23*, Ace1-TAP, and TAP-Δ*fbx23* strains (Fig 5D&5E and S3 Data). In addition, as the transcription of *brlA* gene was downregulated in the Δ*fbx23* mutant, the enrichment of PoAce1 in the promoter of *brlA* gene was also determined.

The detailed strategy for ChIP-qPCR design is shown in Fig 5D&5E (top). ChIP-qPCR results demonstrated PoAce1 binding in the promoter and 5’-CDS of the *abrB* gene in the Ace1-TAP strain. Enrichment of PoAce1 was observed at all assayed regions (R1/2/3/5/6). However, despite the PoAce1 protein accumulating in the TAP-Δ*fbx23* strain, PoAce1 binding of the target gene was defective; the levels of PoAce1 binding DNA at all assayed regions (R1/2/3/5/6) of the *abrB* gene reduced remarkably in TAP-Δ*fbx23* compared with those in the Ace1-TAP strain (Fig 5D, bottom). PoAce1 was somewhat enriched in two (R3/4) of the six detected regions of the *brlA* gene (Fig 5E, bottom). ChIP-qPCR results showed that the PoFbx23 is essential for PoAce1’s binding DNA activity under glucose signal. PoAce1 binds to the regions adjacent to the core promoter of the target gene within the normal cells; the active form of PoAce1 is required in *abrB* expression. In the absence of PoFbx23, despite PoAce1 protein being more stable and accumulating, it no longer binds to the target gene and takes on an inactive form; the expression of the target gene is reduced due to the dysfunctional PoAce1.

### PoFbx23 absence under the cellulose signal results in an accumulation of PoAce1 protein without affecting the Poace1 gene transcription

An individual F-box protein can perform different functions in response to different signals [10]. In cellulase-producing fungi like *T*. *reesei* and *P*. *oxalicum*, Ace1 is of interest as a negative regulator of (hemi)cellulase gene expression under (hemi)cellulose induction signal [26,27]. We investigated how PoFbx23’s absence affected PoAce1 under the cellulose signal.

**Fig 6:**
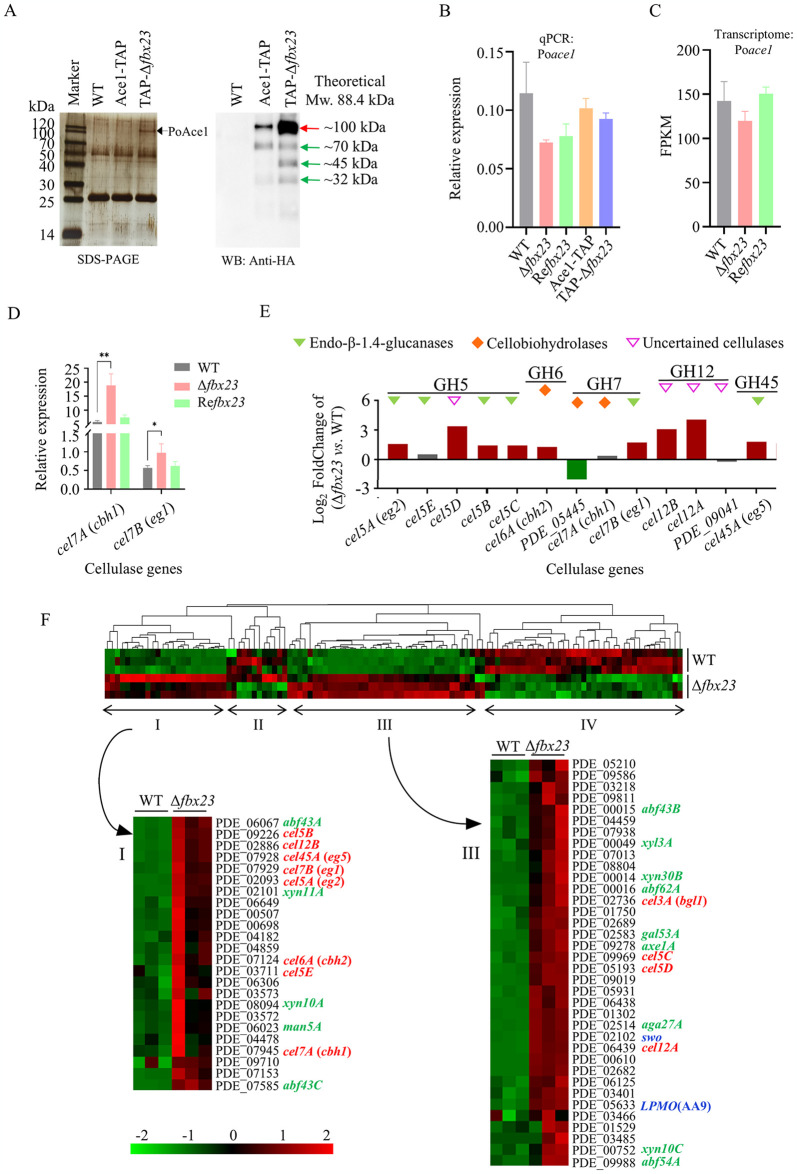
The effect of PoFbx23 on the PoAce1 protein abundance and transcription levels of genes encoding plant cell wall-degrading enzymes under cellulose signal. the strains were grown in VMMC. **(A)** The results of TAP using PoAce1 as bait in the Ace1-TAP and TAP-Δ*fbx23* strains. **Left**, silver staining after SDS-PAGE of TAP eluate. The dark arrow represents the protein PoAce1 (about 100 kDa on the gel, theoretical MW: 88.4 kDa). **Right**, the Western blot of TAP eluate using an anti-HA antibody. **(B)** The transcription level of the Po*ace1* gene in the WT, Δ*fbx23*, Re*fbx23*, SltT-TAP, and TAP-Δ*fbx23* strains, assayed by RT-qPCR. The values were normalized to the *actin* gene level considered 1. **(C)** The transcription level of Po*ace1* gene in the WT, Δ*fbx23*, Re*fbx23* revealed by transcriptome data. The copy number of unambiguous tags mapping to genes was normalized to FPKM. **(D)** Transcription levels of cellulase genes *cbh1* and *eg1* were determined by qRT-PCR. The values were normalized to the *actin* gene level considered 1. **(E)** Transcription levels of 13 cellulase genes in the Δ*fbx23* mutant compared with WT revealed by transcriptome data. **(F)** Clustering analysis of 115 genes that encode plant cell wall-degrading enzymes in the Δ*fbx23* mutant compared with WT using Genesis [56]. The color of each block represents the log_2_ (fold change) in gene transcription of Δ*fbx23 vs*. WT. The genes marked with red fonts are directly related to cellulose degradation. The genes marked with green fonts are directly related to hemicellulose degradation. The genes marked with blue fonts encode the protein/enzyme assisting the breakdown of plant cell walls.

Using the TAP method, PoAce1 was obtained from mycelial cells of the same biomass of WT, Ace1-TAP, and TAP-Δ*fbx23* strains after they were cultured in VMMC liquid. When PoFbx23 is present in the Ace1-TAP strain, PoAce1 exists as a full-length version (~100 kDa, theoretical molecular weight is 88.4 kDa), and several low-molecular-weight degraded versions (about 70-, 45-, and 32-kDa). The abundance of intracellular PoAce1 was significantly increased in the TAP-Δ*fbx23* strain. While the band of PoAce1 was nearly invisible on the SDS-PAGE of the eluate of the Ace1-TAP strain, they were visible at ~100 kDa on the TAP eluate of the TAP-Δ*fbx23* strain (Fig 6A, left). The Western Blot results also demonstrated a stronger PoAce1 signal at about 100-, 70-, 45-, and 32-kDa of the TAP-Δ*fbx23* strain than that of Ace1-TAP (Fig 6A, right). The results show that PoAce1 protein is more stable after the deletion of Po*fbx23*; PoAce1 protein is accumulated in the cell under cellulose signal.

Initially, we hypothesized that the increase in PoAce1 protein abundance was due to the increased transcription of its genes. Therefore, RT-qPCR was used to measure the transcript levels of the Po*ace1* gene in WT, Δ*fbx23*, Re*fbx23*, Ace1-TAP, and TAP-Δ*fbx23* grown in VMMC liquid. It was found that neither the Δ*fbx23* mutant nor the TAP-Δ*fbx23* mutant showed a significant change in the transcript levels of the Po*ace1* gene compared to their respective parent strains, the WT and Ace1-TAP (Fig 6B). Additionally, the transcriptome data of WT and Δ*fbx23* grown in VMMC also showed that the transcription levels of Po*ace1* gene were not significantly different (Fig 6C). The results demonstrate that the deletion of Po*fbx23* does not affect Po*ace1* gene transcription but results in the accumulation of PoAce1 protein under cellulose signal, which is the same observation as under glucose signal (Fig 5).

### PoFbx23 absence results in increased transcription levels of the (hemi)cellulase gene in response to cellulose induction signal

The absence of PoFbx23 results in an accumulation of PoAce1 protein under the cellulose signal. As transcription factor Ace1 has been demonstrated to be a negative regulator for transcription of the (hemi)cellulase gene in various fungi [26–28], it was initially assumed that the expression of (hemi)cellulase genes could be repressed in the Po*fbx23* deletion mutant. However, the RT-qPCR and transcriptome results of (hemi)cellulase genes proved that the initial assumption was wrong. PoFbx23’s absence increases transcription levels of the (hemi)cellulase gene under the cellulose induction signal.

RT-qPCR was used to determine the expression of the two essential cellulase genes *cbh1* (encoding exocellulase/cellobiohydrolase, PDE_07945/Cel7A) and *eg1* (encoding endocellulase/(endo-β-1,4-glucanase, PDE_07929/Cel7B) in the WT and Δ*fbx23* cultivated in VMMC liquid. These two cellulases accounted for more than 70% of all the cellulases secreted by cell cultures in response to cellulose signals [32]. There was a significant increase in both *cbh1* and *eg1* expression in Δ*fbx23* comparison to WT (Fig 6D).

Then, the transcriptome data of WT and Δ*fbx23* were also examined following their cultivation in VMMC liquid. The expression levels of 1332 genes between the WT and the Δ*fbx23* significantly differed (fold change ≥ 2, Q-Value < 0.05) (S2 Spreadsheet, Sheet 3). Among the regulated genes, 780 genes (58.6%) were downregulated, and 552 genes (41.4%) were upregulated in the Δ*fbx23* compared with those in the WT (S2 Spreadsheet, Sheet 3). GO enrichment analysis revealed that genes related to cell wall polysaccharide metabolic/catabolic process, cellulose metabolic/catabolic process, xylan metabolic/catabolic process, and β-glucan metabolic/catabolic process (S3 Spreadsheet, Sheet1 Biological Process), and cellulose binding, monooxygenase activity, polygalacturonase activity, xylanase activity, cellulase activity (S3 Spreadsheet, Sheet2 Molecular Function) were enriched in the genes that were differentially expressed in the Δ*fbx23* compared to the WT. This result suggests that the deletion of Po*fbx23* does affect the gene expression involved in the process of cell wall polysaccharide metabolic/catabolic, specifically the transcription of cellulase and xylanase (hemicellulase) genes.

The transcription levels of all genes encoding cellulases were examined. It is predicted that the *P*. *oxalicum* genome contains 13 cellulases [32]: 5 members of the glycoside hydrolase (GH) 5 family (PDE_02093/*eg2*/*cel5A*, PDE_03711/*cel5E*, PDE_05193/*cel5D*, PDE_09226/*cel5B*, PDE_09969/*cel5C*), one member of the GH6 family (PDE_07124/*cbh2*/*cel6A*), three members of the GH7 family (PDE_05445, PDE_07945/*cbh1*/*cel7A*, PDE_07929/*eg1*/*cel7B*), three members of the GH12 family (PDE_02886/cel12B, PDE_06439/cel12A, PDE_09041), and one member of the GH45 family (PDE_07928/eg5/cel45A). Of the 13 genes, 12 are upregulated in the Δ*fbx23* mutant (Fig 6E red bars), except for only one PDE_05445 downregulated (Fig 6E, green bar).

The transcription levels of genes encoding plant cell wall-degrading enzymes were also examined. The genome of *P*. *oxalicum* is predicted to contain 115 genes that encode plant cell wall-degrading enzymes, which comprise most of the extracellular proteins secreted by the cells under the cellulose signal [32]. The clustering analysis was performed according to the transcription data of 115 genes, identifying 4 co-regulated gene clusters, I/II/III/IV (Fig 6F). The transcription levels of genes in clusters I and III were upregulated, whereas genes in clusters II and IV were downregulated in the Δ*fbx23* mutant. Nearly all of the genes encoding the key exocellulases, endocellulases, hemicellulases, β-glucosidase and lytic polysaccharide monooxygenases (LPMO) that assist the breakdown of plant cell wall are found in Clusters I and III. Cluster I include the key cellulase genes such as cellobiohydrolase genes *cbh1* and *cbh2*, and the endo-β-1,4-glucanase genes *eg1* and *eg2* (Fig 6F cluster I, red fonts), the key hemicellulase genes such as xylanase genes *xyn10A* (PDE_08094) and *xyn11A* (PDE_02101) as well as the xylosidase gene *xyl3A* (PDE00049) and the mannosidase gene *man5A* (PDE06023) (Fig 5C cluster I, green fonts). Cluster III also includes cellulase genes such as endo-β-1,4-glucanase genes *cel5C* and *cel5D*, β-glucosidase gene *bgl1* (Fig 6C cluster III, red fonts), some hemicellulase genes (Fig 6C cluster III, green fonts), and LPMO gene (PDE_05633) and swollenin gene (PDE_02102) which assist the breakdown of plant cell wall (Fig 6C cluster III, blue fonts) (Fig 6F).

The results of RT-qPCR and transcriptome support a negative correlation between the regulatory function of PoFbx23 protein and the transcription of (hemi)cellulase genes. Since (hemi)cellulase synthesis is usually controlled at the transcriptional level, increased transcription of the (hemi)cellulase gene in the Δ*fbx23* mutant may result in a rise in (hemi)cellulase synthesis. Fungal (hemi)cellulases are secreted extracellularly. The filter paper activity (FPA) indicates the synthesis of total cellulase; xylanase activity indicates the synthesis of main hemicellulase, and carboxymethyl cellulose activity (CMCase) indicates the synthesis of endocellulase (endo-β-1,4-glucanase). These enzymatic activities were determined for WT, Δ*fbx23*, and Re*fbx23* incubated in VMMC liquid for 2 to 6 days. There was a significant increase in FPA, xylanase activity, and CMCase activity in the Δ*fbx23* compared to the WT. For example, on the fourth day of cultivation, the FPA, xylanase activity, and CMCase activity of Δ*fbx23* increased by 39.2%, 64.8%, and 16.9% compared to WT, respectively (S3G~I Fig). Cellulases and hemicellulases account for a large proportion of the extracellular proteins of *P*. *oxalicum* [32]. An assay of the extracellular protein concentration the Δ*fbx23*’s extracellular proteins showed a significantly higher protein content than the WT (S3F Fig). This result also supports the higher ability of the Δ*fbx23* to synthesize cellulase and hemicellulase. In addition, the Δ*ace1* mutant strain showed increased levels of protein secretion and (hemi)cellulase production (S3F~I Fig). The results suggested a correlation between PoFbx23 and PoAce1 in regulating (hemi)cellulase production.

### Accumulated PoAce1 under cellulose signal no longer binds the (hemi)cellulase genes

**Fig 7:**
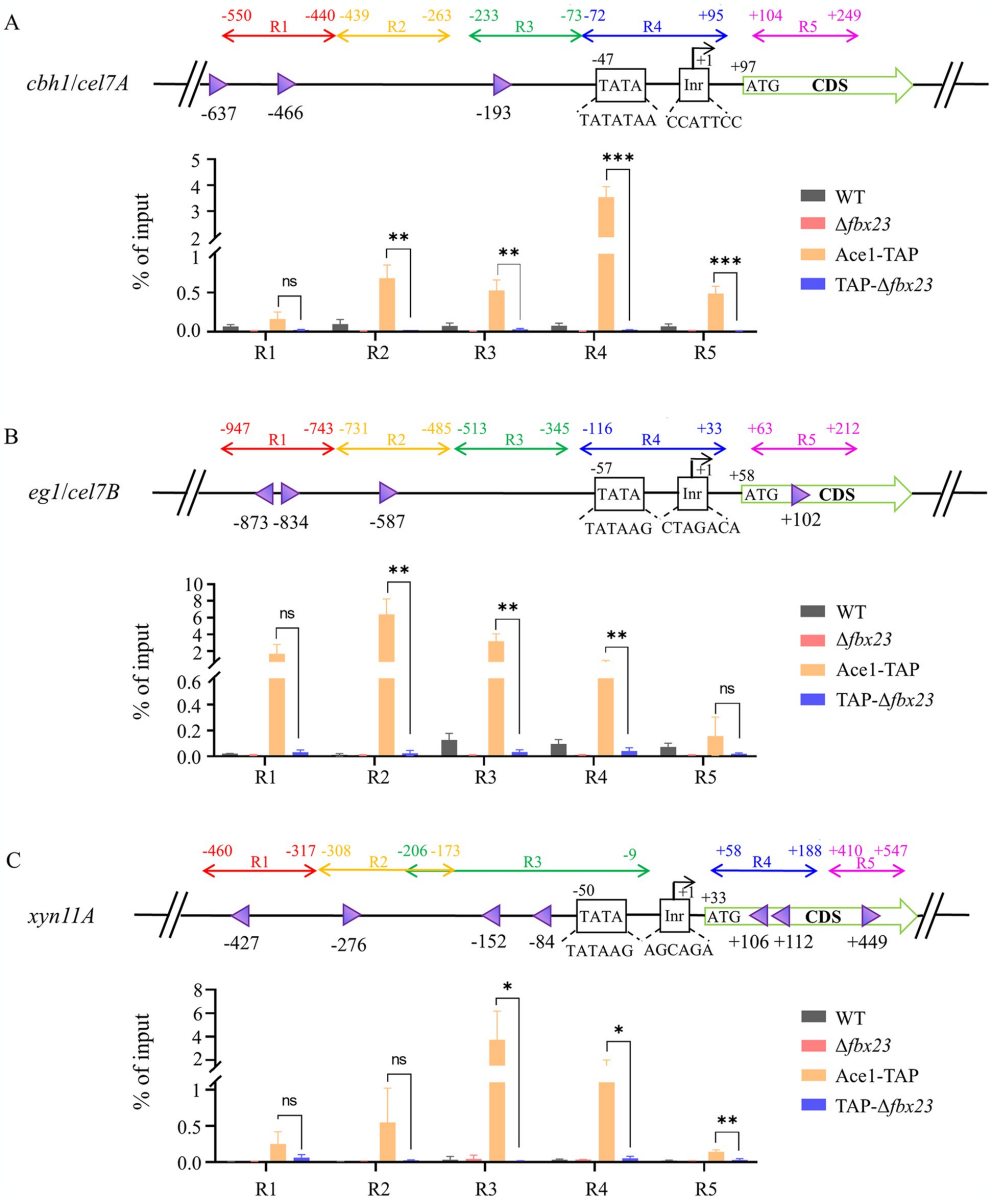
PoAce1 enrichment in the specific regions of (hemi)cellulase genes assayed by ChIP-qPCR. The strains were grown in VMMC. **(A)** Cellulase (cellobiohydrolase) gene *cbh1* **(B)** Cellulase (endo-β-1,4-glucanase) gene *eg1* **(C)** Hemicellulase (xylanase) gene *xyn11A*. **Top subgraphs**, Strategies of ChIP-qPCR for each gene. The TSS is designated as +1. The initiator (Inr) and TATA box are illustrated. For genes *cbh1* and *eg1*, five specific regions (R1 to R5) of each gene were designed for qPCR assay. Region 1 (R1), region 2 (R2) and region 3 (R3) are located upstream of the TSS. Region 4 (R4) covers the TATA-box and the Inr. Region 5 (R5) is located in the 5′ region of the CDS of each gene. For the *xyn11A* gene, R1, R2, and R3 are located upstream of the TSS. R2 and R3 overlap for the appropriate design of primers. No appropriate primers for qPCR could be designed close to the Inr region. R4 is located in the 5′ region of the CDS of *xyn11A*. R6 is located in the middle of the CDS of *xyn11A*. Purple triangles indicate the putative DNA-binding sites of PoAce1. The orientation of the triangle represents the orientation of the binding motif. **Bottom subgraphs**, the results of ChIP-qPCR for each gene. The relative enrichment of IP DNA was calculated by % of input. All values are means from measurements in biological triplicates. The error bars indicate standard deviations, *p < 0.05, **p < 0.01, ***p < 0.001.

The Po*fbx23* deletion strain showed accumulation of PoAce1 protein under cellulose signal but increased transcription of the (hemi)cellulase gene. This finding contradicts earlier research, which indicates that Ace1 is a negative regulator of the transcription of the (hemi)cellulase gene in *P*. *oxalicum* and other cellulase-producing fungi [26–28]. The repressing activity of PoAce1 seems lost due to Po*fbx23* deletion. Then, ChIP-qPCR was used to detect the degree of enrichment of PoAce1 protein in the promoter of the key cellulase gene *cbh1* and *eg1*, and the key xylanase (hemicellulase) gene *xyn11A* in the WT, Δ*fbx23*, Ace1-TAP, and TAP-Δ*fbx23* strains grown in VMMC. The schemes and results of ChIP-qPCR are shown in Fig 7 and S3 Data. Five typical regions (Regions 1–5) that cover upstream sequences and CDS were studied for each gene.

For the ChIP-qPCR design of the *cbh1* gene, regions 1, 2, and 3 (R1/2/3) are located at approximately 600 bp upstream (−) of the TSS. Region 4 (R4) covers the core promoter, including the TATA box and initiator element (Inr). Region 5 (R5) is located proximately at the 5’ region of CDS. ChIP-qPCR results demonstrated PoAce1 binding in Regions 2/3/4 and 5’-CDS in the Ace1-TAP strain. However, despite the PoAce1 protein accumulating in the TAP-Δ*fbx23* strain, PoAce1 binding of the target gene was defective; the levels of PoAce1 binding DNA at various regions (R2-5) of the *cbh1* gene reduced remarkably in the TAP-Δ*fbx23* compared with those in the Ace1-TAP strain (Fig 7A).

The ChIP-qPCR design of the *eg1* gene is similar to that of the *cbh1* gene. ChIP-qPCR results demonstrated that the levels of PoAce1 binding DNA in Regions 2/3/4 reduced remarkably in the TAP-Δ*fbx23* strain compared with those in the Ace1-TAP strain. However, there was no significant difference between the levels of PoAce1 binding to R1 and R5, despite two and one PoAce1 binding sites in R1 and R5, respectively (Fig 7B).

For the ChIP-qPCR design of the *xyn11A* gene, R1 and R2 are located at approximately 500 bp upstream of the TSS. R3 covers the core promoter, including the TATA box. R4 and R5 are located in the 5’ region and middle of the CDS region, respectively. ChIP-qPCR results demonstrated that the levels of PoAce1 binding DNA in regions 3/4/5 reduced remarkably in the TAP-Δ*fbx23* compared with those in the Ace1-TAP strain. However, there was no significant difference between the levels of PoAce1 binding to R1 and R2 (Fig 7C). We noticed that the “percent of input” of PoAce1 enrichment at *cbh1*, *eg1*, and *xyn11A* have the highest values of 3.54, 6.39, and 3.74, respectively, while the “percent of input” of PoAce1 enrichment at *brlA* and *abrB* gene is relatively low, with a maximum of 0.75 and 0.92, respectively. The results suggest that the degree of PoAce1 enrichment in (hemi)cellulase genes is much higher than that of *brlA* and *abrB* genes, indicating PoAce1’s primary direct regulatory role in regulating (hemi)cellulase gene transcription.

ChIP-qPCR results showed that the PoFbx23 protein is essential for PoAce1’s binding DNA activity under cellulose signal. The PoAce1 protein binds to the regions adjacent to the core promoter of the target gene within the normal cells; the active form of PoAce1 works as a repressor to inhibit the transcription of the (hemi)cellulase gene. In the absence of PoFbx23, despite PoAce1 accumulating, it no longer binds to the target genes and takes on an inactive form; the transcription of the (hemi)cellulase gene is derepressed due to the dysfunctional PoAce1.

### PoAce1 is a ubiquitinated protein and takes the UPS pathway

The absence of PoFbx23 resulted in the accumulation of PoAce1. It is necessary to detect whether PoAce1 takes the UPS pathway.

**Fig 8:**
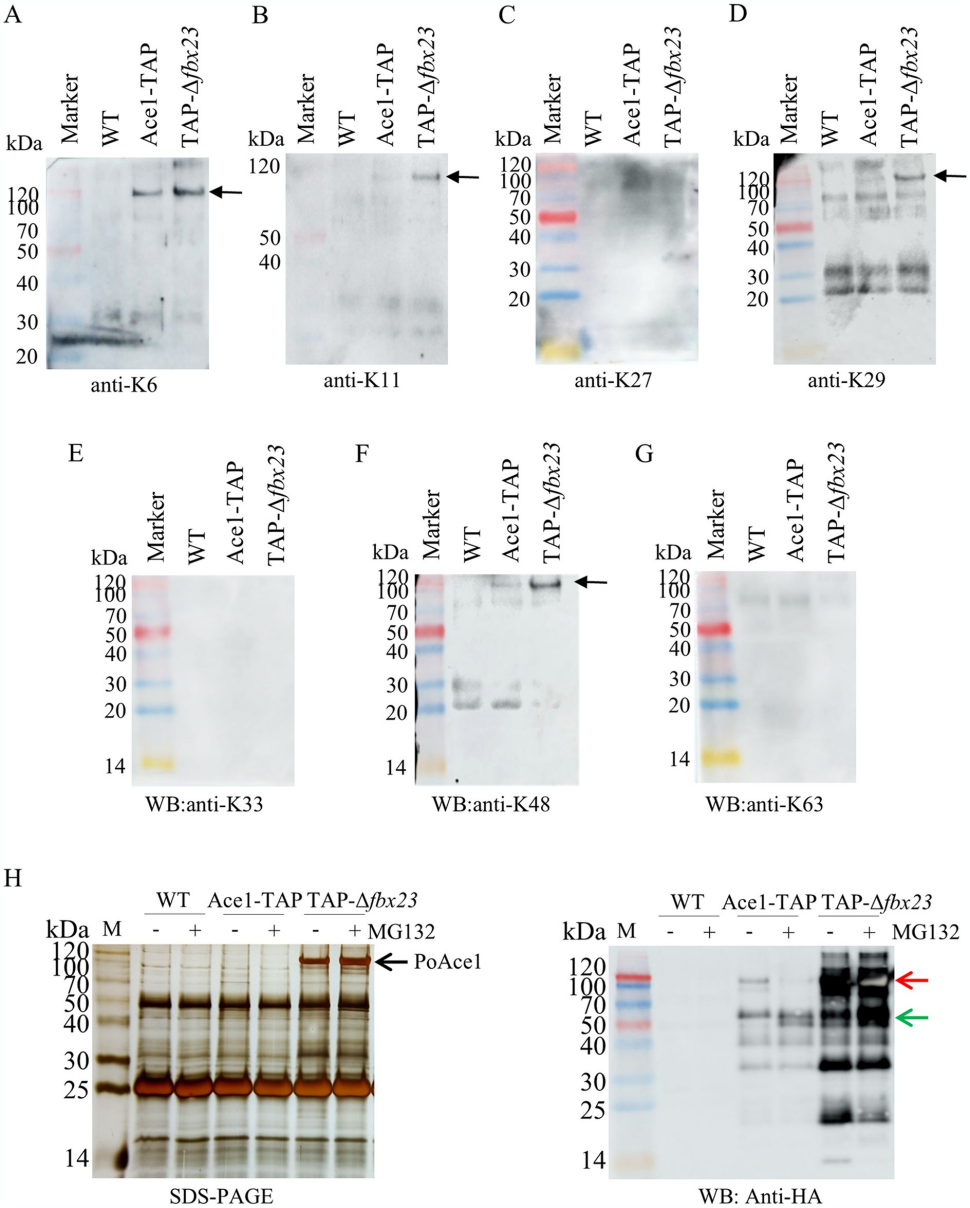
Ubiquitination of PoAce1 and effect of proteasome inhibitor MG132 on PoAce1. **(A~G)** The ubiquitination modification of PoAce1 in WT, Ace1-TAP, and TAP-Δ*fbx23*, detected using anti-K6-, K11-, K27-, K29-, K33-, K48-, and K-63 linkage specific polyubiquitin antibodies, respectively. The dark arrows represent the protein PoAce1 (about 100 kDa on the gel, theoretical MW: 88.4 kDa). **(H)** The results of TAP using PoAce1 as bait in the Ace1-TAP and TAP-Δ*fbx23* strains treated with and without MG132. **Left**, silver staining after SDS-PAGE of TAP eluate. The dark arrow represents the protein PoAce1 (about 100 kDa on the gel, theoretical MW: 88.4 kDa). **Right**, the Western blot of TAP eluate using an anti-HA antibody.

Firstly, we detected whether PoAce1 is a ubiquitinated protein. As substrate proteins are linked to ubiquitin using seven distinct ubiquitin lysine residues (Lys6, Lys11, Lys27, Lys29, Lys33, Lys48, and Lys63), we used anti-K6-, K11-, K27-, K29-, K33-, K48-, and K-63 linkage specific polyubiquitin antibodies to detect the ubiquitination level of PoAce1 (Fig 8). The result showed that PoAce1 has K6-, K11-, K29-, and K48-linked polyubiquitin modification (Fig 8A, 8B, 8D & 8F, black arrows), but without K27-, K33-, and K63-polyubiquitin (Fig. 8C, 8E & 8G). All of the polyubiquitin signals are present where the intact PoAce1 protein is (at ~100 kDa), while the degraded versions of PoAce1 do not exhibit any polyubiquitin signals. Then, we assayed the Ace1-TAP and TAP-Δ*fbx23* strains treated with or without proteasome inhibitor MG132. SDS-PAGE showed that the silver-stained bands of PoAce1 were thickened after TAP-Δ*fbx23* was treated with MG132 (Fig 8H, left, black arrow), corresponding to a stronger signal in the Western Blot (Fig 8H, right, red arrow). It was unexpected that the PoAce1 signal at ~100 kDa was attenuated in the MG132-treated Ace1-TAP sample. However, degraded versions (~ 50kDa) of PoAce1 accumulate in both Ace1-TAP and TAP-Δ*fbx23* samples treated with MG132 (Fig 8I, right, green arrow). PoAce1 accumulation was promoted by MG132 treatment, but this effect was weak and significantly less than that caused by PoFbx23 absence. It should be noted that PoAce1 maintains ubiquitin modifications and accumulates with degraded versions even in the absence of PoFbx23. The result gives a hint that PoAce1 may have other UPS besides the SCF^Fbx23^. Indeed, the results of Ace1-TAP show the presence of the protein PoMub1 (Table 2, ranked 6th), a component of the Mub1/Ubr2 ubiquitin ligase complex.

### PoFbx23 mostly takes the form of free protein within cells

The PoFbx23 protein possesses seven copies of WD repeats S1 Fig. As WD repeats are known as a site for protein-protein or protein-DNA interaction and proteins containing WD repeats are known to serve as platforms for the assembly of multiple proteins [42], it is considered that PoFbx23 may recognize other substrate proteins in addition to PoAce1 as its substrate. We constructed the Fbx23-TAP strain to validate the association between PoFbx23 and PoAce1 further and identify additional potential substrates for the PoFbx23. The WT strain containing native PoFbx23 was used as a control. The eluate of the Fbx23-TAP strain was obtained by two-step tandem purification. Then, the eluate was separated into three sections: one for SDS-PAGE, one for Western blot, and one for MS-MS assay to identify proteins with interactions with protein PoFbx23.

**Fig 9:**
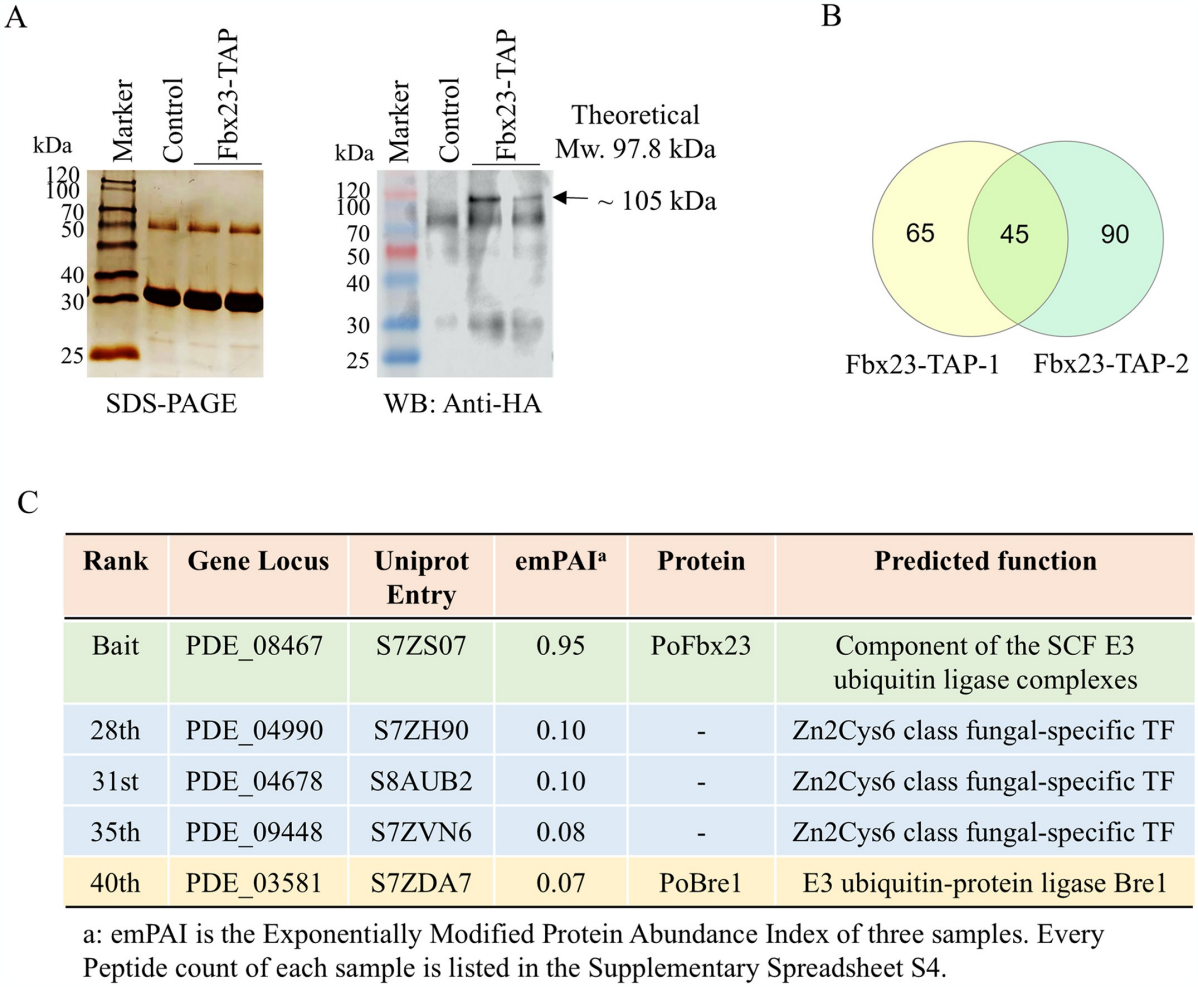
The results of TAP-MS using PoFbx23 as bait. **(A) Left**, silver staining after SDS-PAGE of Fbx23-TAP eluate. **Right**, Western blot of Fbx23-TAP eluate using anti-HA antibody. The arrow represents the protein PoFbx23 (approximately 105 kDa on the gel, theoretical MW: 97.8 kDa). **(B)** Intersection proteins between two Fbx23-TAP biological samples (The detailed information about proteins is shown in S4 Spreadsheet). **(C)** Transcription factors interacting with PoFbx23 identified through TAP-MS. The bait PoAce1 has the highest emPAI (S4 Spreadsheet, sheet 1, row 3, green background). Three Transcription factors, PDE_04990, PDE _04678, and PDE _09448, are racked 28th (row 30), 31st (row 33), and 35th (row 37) in S4 Spreadsheet, sheet 1 (blue background), respectively.

There is no specific band between the control and Fbx23-TAP on SDS-PAGE (Fig 9A, left). However, the Western blot result revealed a signal at about 105 kDa (Fig 9A, right, dark arrow, PoFbx23’s theoretical molecular weight with the TAP tag added is 97.8 kDa). The proteins in the third section of the eluent were analyzed using LC-MS/MS to identify the bait and interacting proteins. The PepCount identified through TAP-MS is listed in S4 Spreadsheet (Sheet 2 & Sheet 3) and S4 Data. The proteins observed in two Fbx23-TAP samples but not in the controls are listed in S4 Spreadsheet (Sheet 1). The proteins were ranked by emPAI [41]. 110 and 135 proteins were identified in two samples of Fbx23-TAP, respectively. 45 proteins were observed in two Fbx23-TAP samples but not the controls (Fig 9B). They are considered reliable proteins with putative interaction with the PoAce1.

As expected, the bait PoFbx23 has the highest emPAI (S4 Spreadsheet, Sheet 1, green background). Interestingly, the TAP results did not reveal the presence of PoSkp1, an adaptor that bridges the core SCF complex with the PoFbx23. This absence could be attributed to multiple FBPs existing in the cell, such as the over 50 FBPs in the genome of *P*. *oxalicum* and over 70 FBPs in *A*. *nidulans* [15]. These FBPs compete with Fbx23 for binding to SKP1. In the research about *A*. *nidulans* AnFbx23, AnSkp1 (AN2302) was pulled down by AnFbx23 only under the condition of H_2_O_2_ oxidative stress but not detected under the condition of normal or NaCl osmotic stress [15]. This finding suggests that most Fbx23 proteins are free forms within the cell, as there is competition among the numerous F-box proteins in cells for binding to Rbx1-Cul1-Skp1 scaffolds [23]. In other words, only a small portion of PoFbx23 is assembled into the core SCF to form an SCF^Fbx23^ complex that recognizes substrate PoAce1, while the majority of PoFbx23 does not bind to Skp1 and exists in the free form of SCF-independent.

PoAce1 protein was not found in the MS results. This result is not wildly unexpected. Many previous studies have observed this phenomenon: when a transcription factor is used as bait, a multisubunit protein complex, such as a cofactor that interacts with that transcription factor, can be identified in the TAP results. Conversely, the transcription factor is not always found when a specific protein complex subunit is used as the bait protein [36,57]. The low abundance of transcription factors is one possible explanation for this phenomenon. Another possibility is that when a specific subunit is used as bait, a variety of other proteins compete with the transcription factor to bind the subunit because the subunit often contains multiple domains for protein-protein interaction. However, in the research about *A*. *nidulans* AnFbx23, 23 proteins were pulled down as potential interacting proteins with AnFbx23, including AnAce1 [15]. The finding also indirectly supported our result that the PoAce1 is the direct substrate of the SCF^Fbx23^ complex.

Among the 44 potential interacting proteins, three transcriptional regulators were identified. All three transcription factors PDE_04990 (UniProt: S7ZH90), PDE_04678 (UniProt: S8AUB2), and PDE_09448 (UniProt: S7ZVN6) are Zn2Cys6 class fungal-specific transcription factors (S4 Spreadsheet, Sheet1, row 30, 33 and 37) (Fig 9C). Particularly, PDE_04678 was also found in one sample of PoAce1-TAP (S1 Spreadsheet, Sheet 1, row 480), suggesting a very high probability that this transcription factor serves as another actual substrate for PoFbx23 in addition to PoAce1. Interestingly, another E3 ubiquitin-protein ligase Bre1 homolog, (PDE_03581 (UniProt: S7ZDA7), was observed in the Fbx23-TAP result (Fig 9C).

## Discussion

One underdeveloped area of FBP biology is examining the substrates and specificity of binding FBPs, as an individual FBP may govern the degradation of multiple substrates in response to diverse signals [58]. In this study, it was found that the F-box containing PoFbx23 protein recognizes transcription factor PoAce1 as a substrate.

**Fig 10:**
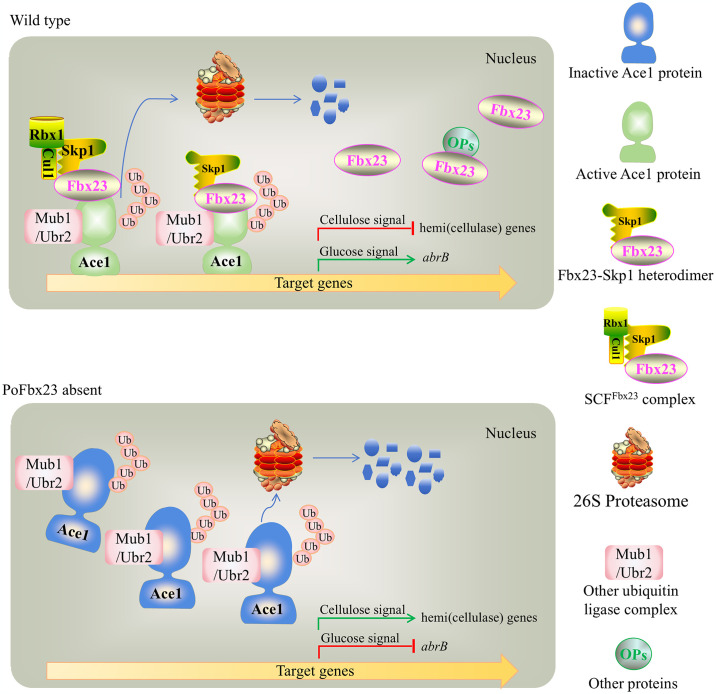
The model in which the PoFbx23 is required in activating PoAce1 to control the expression of target genes. The detailed description of the model is in the main text.

As a sequence-specific transcription factor, the ability to recognize and bind to specific regions of DNA is a fundamental requirement for its activity. This finding underscores the vital role of PoFbx23 in maintaining PoAce1’s activity. By integrating the discoveries from this study with the wealth of knowledge from previous research on the Fbx23 protein and the transcription factor SltA/Ace1 in *P*. *oxalicum* and other fungi [26–31], we propose a model about PoFbx23-Ace1 regulating gene transcription (Fig 10).

In the WT strain, PoFbx23 is found in the nucleus in three different states: (1) most of the PoFbx23 protein is free. Some PoFbx23 proteins can also interact with other proteins, such as transcription factors other than PoAce1; (2) a small amount of PoFbx23 combines with PoSkp1 to form the PoFbx23-Skp1 heterodimer and recognizes the PoAce1; (3) a still smaller amount of PoFbx23 assembles with the core SCF scaffolds (Skp1-Cul1-Rbx1) to form the SCF^Fbx23^ complex. The SCF^Fbx23^ complex and/or the Mub1/Ubr2 ubiquitin ligase complex recognize the PoAce1 as the substrate and lead to the polyubiquitination of PoAce1. The PoAce1 becomes the active form that can bind target DNA due to heterodimer PoFbx23-Skp1 binding or the SCF^Fbx23^ complex binding. Under the glucose signal, the active form of PoAce1 binds to the promoter region of the target gene and activates the target gene (e.g., pigmentation-related gene *abrB*) transcription as a positive regulator. Under the cellulose signal, the active form of PoAce1 also binds to the promoter regions of the hemi(cellulase) genes and represses the target gene transcription as a negative regulator. During this process, some ubiquitinated PoAce1 is coupled with proteasomal degradation by 26S proteasome (Fig 10, top).

In the absence of PoFbx23, PoAce1 accumulates in the cell as the UPS mediated by the SCF^Fbx23^ complex is interrupted. Nevertheless, PoAce1 is still ubiquitination modified and degraded because UPS, mediated by the other ubiquitination system, is still present. Meanwhile, due to the absence of PoFbx23, the accumulated PoAce1 is inactive; and unable to bind to the target gene (e.g., pigmentation-related gene *abrB*) as the positive regulator under the glucose signal. The transcription of the target *abrB* gene is deactivated. Under the cellulose signal, the accumulated inactive PoAce1 cannot bind to the target genes as the negative regulator. The target hemi(cellulase) gene transcription is derepressed (Fig 10, bottom).

From yeast to plants to mammalian cells, F-box proteins have been found to play a role as coactivators of transcription factors. The F-box protein Dsg1/Mdm30 is a transcriptional coactivator that stimulates transcription factor Gal4 turnover in yeast [59]. The *Arabidopsis* F-box protein UFO, a component of the SCF complex, acts as a transcriptional coactivator to regulate floral development by physically interacting with the transcription factor LFY [60]. Fbw1a, also a component of the SCF complex, together with another transcriptional coactivator p300, enhance the transcriptional activity of beta-catenin in mammalian cells [61]. It is evident that PoFbx23 recognition and binding to PoAce1 is necessary for PoAce1 to have DNA-binding activity, although we have not been able to determine whether PoAce1 exhibits its active form due to the binding of the PoFbx23-Skp1 heterodimer and/or the SCF^Fbx2^ complex. Therefore, we propose that PoFbx23 is the transcriptional coactivator of PoAce1.

The ubiquitin ligase SCF complex plays a crucial role in gene transcription by influencing the activity and stability of transcription factors through two pathways: proteasome-dependent (proteolytic) and proteasome-independent (nonproteolytic) [4]. For instance, mammal nuclear factor-kappaB activation represents a paradigm for controlling the function of a transcription factor via proteolysis; NF-kappaB precursors are processed into mature active forms of smaller proteins by the UPS [62]. However, yeast ubiquitin ligase SCF^Met30^-mediated ubiquitination of transcription factor Met4 does not induce Met4 degradation by the proteasome but directly inhibits its activity as a transcriptional activator [63].

In *A*. *nidulans*, three forms (a full-length, a 78-kDa, and a 32-kDa form version) of AnAce1 were observed in cells. The processed 32-kDa form was considered the active form for transcriptional regulation [64]. Upon discovering that the ~32-kDa form of PoAce1 was also present in the Ace1-TAP eluate (Figs 1C, 4A, and 5A, green arrow), our initial hypothesis was that SCF^Fbx23^-Ace1 took the proteasome-dependent pathway: PoAce1 was degraded by SCF^Fbx23^-mediated UPS and took the ~32-kDa active form that regulates the transcription of the target genes; the 32-kDa form would vanish if Po*fbx23* gene is deleted. However, PoFbx23’s absence only resulted in a significant increase in PoAce1 protein abundance relative to normal cells; no discernible difference in PoAce1 protein degradation was observed: every signal of PoAce1 protein, including the full-length (signal at ~ 100 kDa) or degraded versions (signals at low Mw. in Figs 5 & 6, right, green arrows) present in normal cells is detected in the PoFbx23 deletion strain, whether under glucose signal or cellulose signal. Therefore, even though the accumulated degraded versions of PoAce1 in the PoFbx23 deletion strain can support the UPS, the lone presence of ubiquitinated PoAce1 and its degraded versions may not be the active forms of PoAce1; PoAce1 must interact with PoFbx23 to become an active form that can bind to target genes.

We have not demonstrated that PoAce1 is destroyed by Ub-mediated proteolysis, while the overwhelming majority of transcription factors studied to date are destroyed by this pathway [59]. Interestingly, PoMub1, a component of the Ubr2/Mub1 ubiquitin ligase complex, is also observed in the results of Ace1-TAP (Table 2). Yeast Ubr2/Mub1 ubiquitin ligase complex was proved essential for transcription factor Rpn4 ubiquitylation and marked the Rpn4 for degradation [65,66]. The findings raise the possibility that the Mub1/Ubr2 ubiquitin ligase complex is responsible for the ubiquitination of PoAce1 besides the SCF^Fbx23^ complex. They also imply that quality control of transcription factors may not be confined to just one ubiquitin ligase system.

The absence of the PoFbx23 leads to the downregulation of genes associated with fungal asexual development and transcription of secondary metabolite biosynthesis genes under glucose signal and the upregulation of (hemi)cellulase genes under cellulose signal. There could be two reasons for this phenomenon. The dysfunction of PoAce1 is the first explanation, as PoAce1 is a positive regulator for fungal growth and asexual development [29–31] and a negative regulator for (hemi)cellulase genes [26–28]. Along with the PoFbx23 and PoSkp1 of the SCF complex, PoAsh1 (PDE01581), a histone methyltransferase whose protein abundance ranks fifth in the TAP-MS result of Ace1-TAP also caught our attention. Ash1 localizes in the nucleus and is responsible for mono- and dimethylation of histone H3 lysine 36 (H3K36me1 and H3K36me2) [67]. *P*. *oxalicum* H3K36me1 is a repression marker for (hemi)cellulase gene expression [43]. A recent study has shown that *T*. *reesei* Ace1 interacts with protein methyltransferase TrSAM and inhibits cellulase gene expression [68]. Activated PoAce1 by SCF^Fbx23^ may inhibit the expression of cellulase genes by recruiting PoAsh1. FBPs have also been shown to recruit histone methyltransferases in filamentous fungi. The FBP DCAF26 of *N*. *crassa* recruits histone methyltransferase DIM-5 and regulates H3K36 methylation to perform its epigenetic function [69]. Given the high abundance of PoFbx23 proteins in the TAP-MS result of PoAce1 (Table 2), some of the proteins in the TAP-MS result may have interactions with PoFbx23 in addition to interacting with PoAce1 protein. Whether PoAsh1 is recruited by PoAce1 or is targeted for ubiquitination by PoFbx23 remains to be addressed in future studies.

In addition to the dysfunctional PoAce1 as the first explanation, we cannot exclude the second explanation—the role of PoFbx23 itself, as well as the role of PoFbx23 with other substrates, because losing a fungal F-box protein can result in a pleiotropic phenotype, especially when the FBP has multiple targets [16]. The substrate targets of FBPs can be transcription factors, enzymes in metabolic pathways, structural proteins, and inhibitors and/or activators of various biological processes [70]. There are at least 10 known targets (Sic1, Swi5, Far1, Cdc6, Cbl6, Tec1, Gcn4, Hac1, Rcn1, and Ctf13) for the FBP Cdc4 in yeast, and the nonsense mutation of Cdc4 is lethal to yeast [69]. The involvement of PoFbx23 in the plethora of biological processes suggested that the PoFbx23 protein may have other substrates in addition to the transcription factor PoAce1. PoFbx23 has 44 proteins that have putative interactions with it, as revealed by the results of PoFbx23-TAP, including three Zn2Cys6 fungal-specific transcription factors (S4 Spreadsheet). One of the transcription factors, PDE_04990, is the homolog of *S*. *cerevisiae* Asg1. *A*. *nidulans* AnFbx23 has potential interaction with 23 proteins in normal condition, including transcription factors AnAce1 and Taf30, and LaeA-like methyltransferase LlmB (VipC) [15]. Among these putative interacting proteins, there may be false positives as well as real targets of the Fbx23 protein. *S*. *cerevisiae* Asg1 and *A*. *nidulans* VipC have been reported to be involved in fungal growth and development [71,72]. Suppose PoFbx23 does recognize substrates other than PoAce1; in that case, the phenotype caused by the deletion of Po*fbx23* may be due to the superimposed effect of the inactivation of PoAce1 and modulation of other substrates. Furthermore, FBPs are not just involved in simple binding interactions with other proteins, as FBPs may also possess specific and intrinsic enzymatic activities of their own; about 12% of FBPs have activities beyond ubiquitination [23].

The evolution of an FBP is constricted by the requirement to recognize diverse targets. Some FBP-substrate pairs are conserved. For example, the FBP Met30 and its substrate Met4 (Met30-Met4 pair) are evolutionarily conserved in fungi such as budding yeast *S*. *cerevisiae*, fission yeast *S*. *pombe*, *N*. *crassa*, and *A*. *nidulans* [16]. Given the direct evidence from our study that PoAce1 is a substrate for PoFbx23 and the results of that *A*. *nidulans* AnAce1 was discovered in the 23 proteins which were pulled down as potential interacting proteins with AnFbx23 [15], it is reasonable to conclude that Fbx23-Ace1 pair is conserved in *Penicillium* sp. and *Aspergillus* sp.. The discovery of the Fbx23-Ace1 pair can provide help in related research of other filamentous fungi.

## Methods

### Fungal strains and culture conditions

The wild-type (WT) *P*. *oxalicum* 114–2 (CGMCC 5302) was used as the original parent strain. The WT strain and mutants constructed in this study were grown on agar with 10% wheat bran juice at 30°C for 5 days for conidiation. For mycelial growth, the strains were grown at 30°C in 1 × Vogel’s minimal medium (VMM) (50 × Vogel’s salt: 125.0 g, Na_3_Citrate‧2H_2_O, 250.0 g KH_2_PO_4_, 100.0 g NH_4_NO_3_, 10.0 g, MgSO_4_‧7H_2_O, 5.0 g CaCl_2_‧2H_2_O, 0.25 mg biotin, 0.25 g citric acid, 0.25 g ZnSO_4_‧7H_2_O, 0.05 g, Fe(NH_4_)_2_(SO_4_)_2_‧6H_2_O, 12.5 mg CuSO_4_‧5H_2_O, 2.5 mg MnSO_4_‧H_2_O, 2.5 mg H_3_BO_3_, 2.5 mg Na_2_MoO_4_‧2H_2_O, and 1 L of water) [50], plus 2% glucose (w/v) (VMMG) or 2% ball-milled cellulose (VMMC) as a sole carbon source. 1.5% agar was added to the VMMG or VMMC for solid culture.

### Construction of different mutants in *P*. *oxalicum*

All primers used in this study are listed in S2 Table.

The construction strategy and verification for the Ace1-TAP strain, which has PoAce1 fused with a FLAG-HA tag at the C-terminus, are shown in S4A~C Fig. For constructing the Ace1-TAP strain, primer pairs Ace1-TAP-UF/Ace1-TAP-UR and Ace1-TAP-DF/Ace1-TAP-DR were used to amplify the 5’- upstream and 3’-downstream homologous arms of the Po*ace1* gene using *P*. *oxalicum* WT genomic DNA as the template. Primer pairs hph-F/hph-R were used to amplify the marker gene hygromycin B (*hph*) from the plasmid Psilent1 [73]. Then, the three PCR fragments (5’—upstream and 3’ downstream regions of the Po*Ace1* gene and *hph*) were fused with fusion PCR. The PCR product was then amplified using nested primers Ace1-TAP-CSF/Ace1-TAP-CSR and was transformed into the WT to obtain the Ace1-TAP mutant. The Fbx23-TAP strain was constructed using the same strategy as the Ace1-TAP strain. Briefly, primer pairs Fbx23-TAP-UF/Ace1-TAP-UR and Fbx23-TAP-DF/Ace1-TAP-DR were used to amplify the 5’- upstream and 3’- downstream homologous arms of the Po*fbx23* gene. 5’—upstream and 3’ downstream regions of the Po*fbx23* gene and *hph* were fused and amplified using nested primers Fbx23-TAP-CSF/Fbx23-TAP-CSR. The fused product was transformed into the WT to obtain the Fbx23-TAP mutant. The proper fusion of protein PoAce1 or PoFbx23 with the FLAG-HA tag was verified by diagnostic PCR and DNA sequencing.

The construction strategies and verification for the Δ*fbx23* mutant (the gene Po*fbx23* was deleted) and Re*fbx23* mutant (re-complement of the Po*fbx23* gene in the Δ*fbx23* mutant) are shown in S4D~G Fig. For constructing Δ*fbx23* strain, primer pairs Δfbx23-UF/Δfbx23-UR and Δfbx23-DF/Δfbx23-DR were used to amplify the 5’- upstream and 3’- downstream homologous arms of the Po*fbx23* gene using *P*. *oxalicum* WT genomic DNA as the template. Primer pairs hph-F/hph-R were used to amplify the marker gene *hph* from the plasmid Psilent1. Then, the three PCR fragments (5’—and 3’ -flanking regions of the Po*ace1* gene and *hph*) were fused with fusion PCR. The fused product was then amplified using nested primers Δfbx23-CSF/Δfbx23-CSR and was transformed into the WT to obtain the Δ*fbx23* mutant. For constructing the Re*fbx23* strain, primer pairs REfbx23-UF/Refbx23-UR were used to amplify the fragment from 5’- upstream of the Po*fbx23* gene to Po*fbx23* gene terminator using *P*. *oxalicum* WT genomic DNA as the template. Primer pairs ptrA-F/ptrA-R were used to amplify the marker gene pyrithiamine hydrobromide (*ptrA*) from plasmid pME2892 [74]. Then, the two PCR fragments were fused using primers Refbx23-UF/ptrA-R. The fused product was transformed into the Δ*fbx23* mutant to obtain the Re*fbx23* mutant. The mutants Δ*fbx23* and Re*fbx23* were verified by diagnostic PCR and Southern blot.

The construction strategies for the Fbx23-GFP mutant (the protein PoFbx23 fused with a green fluorescent protein (GFP)) and Ace1-GFP mutant (the protein PoAce1 fused with GFP) are shown in S5A&B Fig. For constructing the Fbx23-GFP strain, primer pairs fbx23-GFP-UF/fbx23-GFP-UR and fbx23-GFP-DF/fbx23-GFP-DR were used to amplify the 5’- upstream and 3’- downstream homologous arms of the Po*fbx23* gene using *P*. *oxalicum* WT genomic DNA as the template. Primer pairs gfp-F/gfp-R were used to amplify the *gfp* gene from the plasmid pEGFP. Primer pairs hph-F/hph-R were used to amplify the marker gene *hph* from the plasmid Psilent1. Then, the four PCR fragments (5’- upstream and 3’- downstream of the Po*fbx23* gene, *gfp*, and *hph*) were fused with fusion PCR. The PCR product was then amplified using nested primers fbx23-GFP-CSF/fbx23-GFP-CSR and was transformed into the WT to obtain the Fbx23-GFP strain. Similar to the construction of the Fbx23-GFP strain, primer pairs Ace1-TAP-UF/Ace1-GFP-UR and Ace1-TAP-DF/Ace1-TAP-DR were used to amplify the 5’- upstream and 3’- downstream homologous arms of the Po*Ace1* gene. Then, the four PCR fragments (5’- upstream and 3’- downstream of the Po*Ace1* gene, *gfp*, and *hph*) were fused with fusion PCR. The PCR product was then amplified using nested primers Ace1-TAP-CSF/Ace1-TAP-CSR and was transformed into the WT to obtain the Ace1-GFP strain. For constructing the PoAce1 protein fused the GFP in the Δ*fbx23* background, primer pairs Δfbx23(Ace1-GFP)-UF/Δfbx23(Ace1-GFP)-UR were used to amplify the fragment from 5’- upstream to terminator of the Po*fbx23* gene using *P*. *oxalicum* WT genomic DNA as the template. Primer pairs Δfbx23-UF/Δfbx23(Ace1-GFP)-UR and Δfbx23(Ace1-GFP)-DF/Δfbx23-DR were used to amplify the 5’-upstream and 3’-downstream homologous arms of the Po*fbx23* gene. Primer pairs ptrA-F/ptrA-R were used to amplify the marker gene *ptrA* from plasmid pME2892 [74]. Then, the three PCR fragments (5’- and 3’-flanking regions of the Po*fbx23* gene and *ptrA*) were fused with fusion PCR. The fused product was then amplified using nested primers Δfbx23-CSF/Δfbx23-CSR and was transformed into the Ace1-GFP to obtain the ΔF-Ace1-GFP mutant. The proper fusion of protein PoFbx23 or PoAce1 with GFP was verified by diagnostic PCR and DNA sequencing.

The construction strategy for the Ace1-YFP-Fbx23 mutant (the strain for bimolecular fluorescence complementation (BiFC)) is shown in S5C&D Fig. BiFC strains were constructed through a previously described method [36]. The plasmids pMD18-T-NYFP carrying the coding sequence of the N-terminus (1–155 aa) of yellow fluorescent protein (YFP) and pUC19-CYFP carrying the coding sequence of the C-terminus (156–238 aa) of YFP were used as the expression vectors for BiFC. The Po*ace1* gene was amplified using primers Ace1-NF/Ace1-NR. The PCR product was inserted into the multiple cloning sites (MCS) of plasmid pMD18-T-NYFP to construct the recombinant vector pMD18-T-NYFP-Ace1, which contains protein PoAce1 fused with the N-terminus of YFP (S5C Fig, left). The Po*fbx23* gene was amplified using primers fbx23-NF/fbx23-NR. The PCR product was inserted into the MCS of plasmid pUC19-CYFP to construct the recombinant vector pUC19-CYFP-Fbx23, which contains protein PoFbx23 fused with the C-terminus of YFP (S5C Fig, right). The recombinant vectors pMD18-T-NYFP-Ace1 and pUC19-CYFP-Fbx23 were linearized and simultaneously transformed into the WT to obtain the Ace1-YFP-Fbx23 mutant. The proper fusion of protein PoAce1 with the N-terminus of YFP and protein Pofbx23 with the C-terminus of YFP was verified by diagnostic PCR and DNA sequencing.

### Phenotypic analysis of *P*. *oxalicum* strains

For observation of colony morphology, 1 μL of fresh spore suspension (10^7^ conidia ml^-1^) was point-inoculated onto VMMG agar and then cultivated at 30°C. The colony diameters were measured every day for 9 days. To count the spore yield of different mutants, we coated 150 μL of fresh spore suspension (10^7^ conidia ml^-1^) evenly onto VMMG agar. After cultivated at 30°C for 5 days, 10-mm diameter agars were taken from each plate, and spores were washed with 2 mL of physiological saline containing 0.2% tween 80 by vortexing. Then, the spores were counted using a hemocytometer. The biomass was determined by the dry weight method described previously [75].

### Phylogenetic analysis and domain architecture analysis

The amino acid sequences of Fbx23 homologous proteins from different species were obtained from the UniProt database (https://www.uniprot.org/). The Clustal X and MEGA 7.0 software were used to construct multiple sequence alignments and construct a phylogenetic tree with the neighbor-joining method. The SMART database (https://smart.embl.de/) was used for domain analysis of proteins. The domain architecture patterns were constructed in proportion to the corresponding protein sequences.

### Tandem affinity purification

Fresh conidial suspensions of the *P*. *oxalicum* WT and the TAP strains were inoculated in 2 L of VMMG liquid at 30°C and 180 rpm for 48 h. The mycelia were filtered through miracloth and washed twice with 0.96% NaCl (w/v) containing 1% DMSO and 1 mM PMSF. Afterward, the mycelia were ground in liquid nitrogen. We used about 40 g of ground mycelia per tandem affinity purification and mass spectrometry (TAP-MS) system. Each treatment for TAP-MS was performed in three biological replicates. The TAP-MS procedure was performed as previously described [36]. Briefly, Protein lysis buffer (0.9 g NaCl, 1 M Tris-HCl, pH 7.5, 10 mL glycerol, 0.1 mL NP40, and 0.05 mL protease inhibitor per 100 mL) was used to extract the total protein. Ezview TM Red ANTI-FLAG M2 Affinity Gel (SIGMA, USA) was used for the first affinity purification step. 500 μL 3 × FLAG peptide (150 ng/μL) was used to compete with the ANTI-FLAG M2 affinity resin to obtain the first protein eluate. ANTI-HA resin (SIGMA, USA) was used for the second affinity purification step. Finally, 80 μL of 8 M urea was used to elute ANTI-HA resin to obtain the final protein eluate. The final eluate was separated into three sections following two affinity purification steps. One part of the eluate was assayed with 12.5% SDS-PAGE, followed by silver staining. One part of the eluate was assayed with Western blot using rabbit anti-HA as an antibody (ABclonal, China). The other part of the eluate was assayed through LC-MS/MS for protein identification (APT, Shanghai, China). Rabbit anti-K6-, K11-, K27-, K29-, K33-, K48-, and K-63 linkage specific polyubiquitin antibodies (ABclonal, China) were used to detect the ubiquitin modification of the protein.

### Proteasome inhibitor MG132 treatment

Strain culture and mycelia collection are performed as described in the “Tandem affinity purification” section above. Then, the mycelia are vacuum-infiltrated with 50 μM MG132 (dissolved in DMSO) or DMSO alone for ten minutes and immersed in the same solution for two hours. The target protein was detected as described in the “Tandem affinity purification” section above.

### Microscopy of GFP and BiFC strains

Hyphae of the GFP strains Ace1-GFP and Fbx23-GFP and the BiFC strain Ace1-YFP-Fbx23 were observed under a high sensitivity laser scanning confocal microscope (ZEISS LSM900) (Carl Zeiss, Germany). The nuclei were stained in the dark for 15 minutes by Hoechst 33342 (Sigma, United States). The blue nuclei stained with Hoechst 33342 were observed with excitation light at 405 nm. The green fluorescence of the Ace1-GFP and Fbx23-GFP strains was observed by excitation light at 488 nm. The yellow fluorescence of the Ace1-YFP-Fbx23 strain was observed by excitation light at 488 nm.

### Yeast two-hybrid assay

For yeast two-hybrid assay (Y2H) analysis, the coding sequence (CDS) of two truncated PoAce1, Ace1- (1 to 396 aa) and Ace1- (484 to 816 aa) which avoid DNA binding domain of PoAce1, were amplified by primers A396-ADF/A396-ADR and A816-ADF/A816-ADR, respectively. Then, two CDS were cloned into the plasmid pGADT7 and transformed into *S*. *cerevisiae* Y187. The CDS of PoFbx23 were amplified by primers Fbx23-BDF/Fbx23-BDR, cloned into the plasmid pGBKT7, and transformed into *S*. *cerevisiae* Y2H Gold. Y2H strains were grown on QDO (quadruple-dropout, SD-Ade/-His/-Leu/-Trp) and QDO/x-α-gal/Aba (QDO supplemented with X-α-gal and aureobasidin A) agar to test possible interactions.

### Transcriptome analysis and GO analysis

Fresh spores (10^7^ conidia ml^-1^) were precultured in VMMG liquid at 30°C for 24 h. Equal mycelia was collected through vacuum filtration and was then transferred (0.3 g mycelia/50 mL medium) to VMMG or VMMC. Fresh mycelia of the WT and the Δ*fbx23* after 24-h of cultivation (30°C, 200 rpm) in VMMG or VMMC were harvested and fully ground in liquid nitrogen. Total RNA was extracted with RNAiso Plus reagent (Takara, Japan) and was incubated with 10 U DNase I (Takara, Japan) at 37°C for 30 min to remove the genomic DNA. Transcriptome analysis based on BGISEQ-500 RNA-Seq was performed by the Beijing Genomics Institute (BGI, Wuhan, China). Saturation analysis of each sample indicated their availability for omics analysis (6S Fig). Sequenced reads were mapped against the reference genome of *P*. *oxalicum*. The filtered clean reads for each gene were normalized to fragments per kilobase transcriptome per million mapped reads (FPKM) for differential expression analysis (S5 Data). Significantly different expression between samples were identified through a significance test with combined thresholds (fold change ≥ 2, Q-Value < 0.05). OmicsBox was used for GO annotation and function enrichment analysis with a threshold at FDR ≤ 0.05 [76]. Genesis software was used for Cluster analysis [56]. The raw transcriptome profiling data have been deposited in NCBI’s Gene Expression Omnibus database under the accession number GSE228483.

### Real-time quantitative PCR

Strains were cultivated as previously described in the “Transcriptome Analysis and GO analysis” section. Fresh mycelia after 4-, 24-h of cultivation (30°C, 200 rpm) in VMMG or VMMC were harvested and fully ground in liquid nitrogen. Total RNA of different strains was extracted using RNAiso Plus reagent (Takara, Japan). Genomic DNA removal and cDNA synthesis were performed using RT Reagent Kit With gDNA Eraser (Takara, Japan). The obtained cDNA was used as the template for quantitative PCR. The primers used for RT-qPCR are listed in S2 Table. For each gene, biological triplicates were conducted. Gene expression copy numbers were calculated using the standard curves constructed for each gene, and the data were then normalized with the expression levels of the *actin* gene.

### Cellulase and hemicellulase activity assay

Fresh spores (10^7^ conidia ml^-1^) were precultured in VMMG liquid at 30°C for 24 h. Equal mycelia was collected through vacuum filtration and transferred to VMMC liquid (0.3 g mycelia/50 mL) at 30°C. The culture supernatants from different time points were collected via centrifugation and diluted with 0.2 M NaAc buffer solution (pH 4.8). The activities of filter paper activity (FPA), endo-β-1,4-glucanase (indicated by CMCase activity), and xylanase were assayed following the methods described by Li et al. [27]. Whatman No. 1 filter paper, carboxymethylcellulose sodium salt (CMC-Na) (Sigma-Aldrich, St. Louis, USA), and birch xylan (Ryon, Shanghai, China) were used as substrates. Briefly, 0.5 mL of suitably diluted culture supernatants and 50 ± 1 mg Whatman No. 1 filter paper, or 1.5 mL of 1% (m/v %) CMC-Na or 1% (m/v %) xylan, which was dissolved in NaAc buffer (pH 4.8), were added into 25-mL colorimetric tubes. The mixture was gently stirred and incubated in a 50°C water bath for 30 min. 3 mL 3,5-dinitro salicylic acid (DNS) reagent (10 g 3, 5-dinitro salicylic acid, 20 g sodium hydroxide, 200 g sodium potassium tartrate, 2.0 g redistilled phenol, and 0.5 g sodium sulfite anhydrous per 1000 mL DNS reagent) was then added to stop enzymatic reaction. The tubes were placed in boiling water for 10 min. Finally, 20 mL of distilled water was added and mixed, and the absorbance of the reaction mixture was measured at 540 nm to determine enzymatic activities. One enzyme activity unit was defined as the amount of enzyme required for producing 1 μmol glucose or 1 μmol xylose per minute under the assayed conditions.

### Chromatin immunoprecipitation-quantitative PCR

Strains were cultivated as previously described in the section “Transcriptome Analysis and GO analysis” section. ChIP assays were performed by a previously described protocol [43]. Briefly, the hyphae cultivated in VMMG or VMMC were fixed by adding 1% formaldehyde at 30°C for 10 min with shaking. Then, the glycine was added to obtain a final concentration of 125 mM. The mycelia were filtered by a vacuum pump filter, ground in liquid nitrogen, and mixed in the ChIP-lysis buffer (50 mM HEPES pH 7.5, 150 mM NaCl, 1 mM EDTA, 0.5% Triton X-100, 0.1% sodium deoxycholate, 0.1% SDS, 1 mM PMSF (phenylmethanesulfonyl fluoride), 0.1% protease inhibitor cocktail). Then, the chromatin was sheared into 200~500 bp fragments through sonication. Immunoprecipitation (IP) was performed with an anti-HA antibody (Proteintech, USA) with equal amounts of extracted chromatin (1 mg). The obtained IP products and 0.1 mg input chromatin DNA (without IP) of each sample were subjected to RNase digestion to remove RNA, as well as high heat and proteinase K digestion to reverse crosslinks, followed by phenol-chloroform extraction and ethanol precipitation to get purified IP DNA and input DNA. Then, quantitative PCR was performed. The relative enrichment of IP DNA was defined as the ChIP efficiency and calculated by the input% method as follows: ChIP efficiency = 2−^ΔCt^ × 100%, ΔCt = Ct_IP_ − (Ct_Input_ − log_2_10), where Ct = threshold cycle of PCR. Three biological replicates were established for each sample. Primers used in the RT-qPCR are listed in S2 Table.

## Supporting information

S1 Document The matched peptides of the sliced specific band (Fig 1B, dark arrow) by MS/MS assay after SDS-PAGE. Matched peptides are shown in red fonts.
(PDF)

S1 Table Description of 28 secondary metabolic gene clusters in *P*. *oxalicum*. (DOCX)

S2 Table Primers used in this study. (DOCX)

S1 Fig Phylogenetic and domain architecture analysis of PoAce1 orthologs. **(A)** Phylogenetic analysis of PoAce1 orthologs. Multiple sequence alignment was performed, and a phylogenetic tree was constructed using the maximum likelihood method by Molecular Evolutionary Genetic Analysis (MEGA 7.0). Coefficients were indicated below the respective nodes, and gaps in the alignment were not considered. **(B)** Domain architecture analysis refers to the website SMART (http://smart.embl-heidelberg.de/). The maps were constructed with equal proportions of the respective sequences.
(JPG)

S2 Fig Transcriptome analysis of secondary metabolism gene clusters of the WT and Δ*fbx23* grown in VMMG. The color of each block represents the log_2_(fold change) in gene expression. The red fonts indicate the “backbone” genes predicted in the clusters. **(A)** Cluster 1 **(B)** Cluster 2 **(C)** Cluster 26 **(D)** Cluster 28. The description of 28 secondary metabolic gene clusters is shown in S1 Table. DMAT, Demethylallyl tryptophan synthase; NRPS, Nonribosomal peptide synthetases; PKS, Polyketide synthases; HYBRID, PKS-NRPS hybrid.
(JPG)

S3 Fig The phenotype of the Δ*ace1* and (hemi)cellulase production of various mutants. Colonies **(A)** and colony diameters **(B)** of *P*. *oxalicum* WT and the Δ*ace1* mutant grown on VMMG agar at 30°C. **(B)** Conidia quantification after the WT and the Δ*ace1* mutant grown on VMMG agar at 30°C for five days. Transcription levels of the gene *brlA* **(D**) and *abrB* **(E)** assayed by qRT-PCR after the WT and the Δ*ace1* mutant grown in VMMG liquid at 30°C for 24 hours, respectively. Assay of extracellular protein concentration **(F)**, FPase activity **(G)**, Xylanase activity **(H)**, and CMCase activity **(I)** of the WT the mutants grown in VMMC liquid. The biological triplicates were performed for all enzymatic activity analyses. The mean values and standard deviations were calculated. *p < 0.05, **p < 0.01, ***p < 0.001.
(JPG)

S4 Fig Construction strategy and verification of strains Ace1/Fbx23-TAP, Δ*fbx23*, and Re*fbx23*. **(A)** Construction strategy of Ace1-TAP strain. The construction strategy of the Fbx23-TAP strain is the same as that of the Ace1-TAP strain. **(B)** Results of diagnostic PCR of TAP strains. Lane 1 and lane 2 represent the control *P*. *oxalicum* WT; lane 3 (2555 bp) and lane 4 (3015 bp) represent Ace1-TAP (amplified using primers ace1-TAP-UF/hph-YZR and hph-YZF/ace1-TAP-DR, respectively). **(C)** Sequencing results of the protein PoAce1 fused with the TAP (FALG-HA) tag. **(D)** Construction strategies of strains Δ*fbx23* and Re*fbx23*. **(E)** Results of diagnostic PCR of strains Δ*fbx23* and Re*fbx23*. Lanes 1, 2, and 3 represent the negative control WT; lane 4 (1964 bp). Lane 5 (2122 bp) and Lane 6 (2434 bp) represent Δ*fbx23* (amplified using primers Δfbx23-UF/hph-YZR, fbx23-MYZF/fbx23-MYZR and hph-YZF/Δfbx23-DR, respectively). Lane 7 (4748 bp), lane 8 (4989 bp) and lane 9 (3879 bp) represent Re*fbx23* (amplified using primers Δfbx23-UF/ptrA-YZR, fbx23-MYZF/fbx23-MYZR and ptrA-YZF/Δfbx23-DR, respectively). **(F)** Strategies of the Southern blot. Primers fbx23-sou-F/fbx23-sou-R were used to amplify the probe. **(G)** The results of Southern blot. The theoretical size of WT is 5362 bp; the theoretical size of the Δ*fbx23* mutant strain is 1653 bp; the theoretical size of Re*fbx23* is 5468 bp.
(JPG)

S5 Fig Construction strategies for GFP strains Ace1/Fbx23-GFP and BiFC strain Ace1-YFP-Fbx23. **(A)** Construction strategy of GFP strains Ace1-GFP and Fbx23-GFP. **(B)** Results of diagnostic PCR of GFP strains. Lanes 1, 2, 5 and 6 represent the control WT; lane 3 (2555 bp) and lane 4 (3015 bp) represent Ace1-GFP (amplified using primers Ace1-GFP-UF/hph-YZR and hph-YZF/Ace1-GFP-DR, respectively); lane 7 (2699 bp) and lane 8 (2434 bp) represent Ace1-GFP (amplified using primers Fbx23-GFP-UF/hph-YZR and hph-YZF/Fbx23-GFP-DR, respectively). **(C)** Construction strategy of BiFC strain Ace1-YFP-Fbx23.
(JPG)

S6 Fig Saturation analysis of transcriptome data for the WT and Δ*fbx23*. **(A~C)** The biological triplicates of the WT grown in VMMG. **(D~F)** Biological triplicates of the Δ*fbx23* grown in VMMG. **(G~I)** Biological triplicates of the WT grown in VMMC. **(J~L)** Biological triplicates of the Δ*fbx23* grown in VMMC.
(JPG)

S1 Spreadsheet The result of TAP-MS using PoAce1 as the bait. **(Sheet 1)** The data of proteins interacting with PoAce1 identified through TAP-MS. Row 3, the bait PoAce1; row 4/5/13/75, the components of E3 SCF complex, PoSkp1, PoFbx23, PoRbx1, and PoPoCul1, respectively. **(Sheet 2~4)** The data of biological triplicates Ace1-TAP-1/2/3, respectively. The proteins are ranked by exponentially modified protein abundance index (emPAI).
(XLSX)

S2 Spreadsheet The differentially expressed genes in transcriptome. The differentially expressed genes between the WT and the Δ*fbx23* grown in VMMG **(Sheet 1)** and VMMC **(Sheet 2)**. The differentially expressed genes (fold change ≥ 2, Q-Value < 0.05) between the WT and the Δ*fbx23* grown in VMMC **(Sheet 3)**.
(XLSX)

S3 Spreadsheet GO enrichment analysis of the differentially expressed genes between the WT and the Δ*fbx23* grown in VMMC. **(Sheet 1)** Biological process **(Sheet 2)** Molecular function **(Sheet 3)** Cellular component.
(XLSX)

S4 Spreadsheet The result of TAP-MS using PoFbx23 as the bait. **(Sheet 1)** The data of proteins interacting with PoFbx23 identified through TAP-MS. Row 3, the bait PoFbx23. **(Sheet 2&3)** The data of two biological samples, Fbx23-TAP-1 and Fbx23-TAP-2. The proteins are ranked by exponentially modified protein abundance index (emPAI).
(XLSX)

S1 Data Data that underlies the TAP-MS of three Ace1-TAP samples. (XLSX)

S2 Data Data that underlies the RT-qPCR in this paper. (XLSX)

S3 Data Data that underlies the ChIP-qPCR in this paper. (XLSX)

S4 Data Data that underlies the TAP-MS of two Fbx23-TAP samples. (XLSX)

S5 Data Data that underlies the transcriptome in this paper. The values are fragments per kilobase transcriptome per million mapped reads (FPKM).
(XLSX)
