# Supplementary material for: F-box protein Fbx23 acts as a transcriptional coactivator to recognize and activate transcription factor Ace1
Source: PLoS Genet. 2025 Jan 21;21(1):e1011539. doi: 10.1371/journal.pgen.1011539 (PMC11750091; doi:10.1371/journal.pgen.1011539)
Supplement: S1 Document — Matched peptides are shown in red fonts. (PDF) [file pgen.1011539.s001.pdf]

Mascot Search Results

Protein View

Match to: PDE_01988 Score: 232

Found in search of I:\New folder\BPI_26537_Penicillium_oxalicum_AceA.mgf

Nominal mass (M_r): 89359; Calculated pI value: 5.61

NCBI BLAST search of PDE_01988 against nr
Unformatted sequence string for pasting into other applications

Fixed modifications: Carbamidomethyl (C)
Variable modifications: Gln->pyro-Glu (N-term Q),Oxidation (M)
Cleavage by Trypsin: cuts C-term side of KR unless next residue is P
Sequence Coverage: 9%

Matched peptides shown in Bold Red

```
  1 MASTQFESTR VHPRRRPVVN TSSNLAKTDE EPTASKLQLK KGETFNAPTS
 51 PPSAESDPVM NIRSLPHRSP TSLDALAASE ESMSSIFERL NLDENSEGQG
101 ASTDNQSAGA STLNSAADKD EHNPFPSVKK VSTQQEDHDH DSDSGLGSSV
151 SDCDSMSEIS EQDNHDASVE QDSTITSPIA ASQGTNPRHQ LPLSACKQIE
201 RYLLVPLLKE PKLEDFHPLV RSVPTRIANK QIVCLRDLEK ILLWLAPKFS
251 KSRSSYLSFC EYTIQCLHTS VSHLNAKDQR LPADRPYTNG YFLDLVTQVR
301 RYAAMVQASK AEALQSNKDK PSVPPASLQG GLSVNGCAAE LVIMTDGQVI
351 SLATGKPFEG AVSIKRAHET VDEVTDEGVV RSMARRKKNA PPMDINQKCA
401 HCDKVFKRPC DLTKHEKTHS RPWKCPDTTC KYNLVGWPTE KERDRHVNDK
451 HSENPIIFKC EFGGCTYTSK RESNCKQHME KAHGWKYNRQ KSNSRARGSK
501 SKRGTSLRAA SHQDSPSTPD AMTPASGQTD FNTPSLGPTP SPCEPSLIYS
551 DGSSFINFAD PPAPVPGNGY SAFYNASVVN QPEAAMSYQA SPEGTAPSYQ
601 TPRTYQTSPD ATMAHDFQFT AEDIANLGSL GSLEAQFAMG NPNELVSHLN
651 MHQSIVASMS SVPSASSVPD LSGSVSASQG NSPCAPAASG GNLCDNIDWT
701 RVEYSLQNNI NDLHGGNDNG NDQAMMMSGL SPTAQGHLML FSPDNGLAAA
751 NIGASFPYGG QDMQQNLQDF SGQELQDFTL FETPMPTYPA ANLTDPAFNW
801 VGGPWPGMDK AIAFD
```
