# Supplementary material for: F-box protein Fbx23 acts as a transcriptional coactivator to recognize and activate transcription factor Ace1
Source: PLoS Genet. 2025 Jan 21;21(1):e1011539. doi: 10.1371/journal.pgen.1011539 (PMC11750091; doi:10.1371/journal.pgen.1011539)
Supplement: S1 Table — (DOCX) [file pgen.1011539.s002.docx]

**Table S1 Description of 28 secondary metabolic gene clusters in *P. oxalicum***

| **Cluster** | **Gene ID (locus_tag)** | **SMURF "backbone gene" prediction ^a^** | **Oxidase/** **reductase** |
| --- | --- | --- | --- |
| Cluster_1 | PDE_00787 - PDE_00814 | NRPS (PDE_00789)  NRPS (PDE_00793)  DMAT(PDE_00797)  DMAT(PDE_00807)  NRPS (PDE_00810) | Yes |
| Cluster_2 | PDE_01064 - PDE_01077 | DMAT (PDE_01066)  NRPS (PDE_01071)  NRPS (PDE_01077) | Yes |
| Cluster_3 | PDE_01166 - PDE_01177 | PKS-Like (PDE_01173) |  |
| Cluster_4 | PDE_01185 - PDE_01198 | NRPS-Like (PDE_01185) |  |
| Cluster_5 | PDE_01212 - PDE_01220 | NRPS (PDE_01212) |  |
| Cluster_6 | PDE_01230 - PDE_01242 | PKS (PDE_01235) | Yes |
| Cluster_7 | PDE_01418 - PDE_01233 | NRPS (PDE_01432) | Yes |
| Cluster_8 | PDE_02130 - PDE_02141 | NRPS (PDE_02131)  PKS (PDE_02141) | Yes |
| Cluster_9 | PDE_02203 - PDE_02204 | NRPS-Like (PDE_02203) | Yes |
| Cluster_10 | PDE_02600 - PDE_02610 | NRPS (PDE_02610) | Yes |
| Cluster_11 | PDE_03298 - PDE_03304 | NRPS (PDE_03304) | Yes |
| Cluster_12 | PDE_03415- PDE_03416 | NRPS (PDE_03416) |  |
| Cluster_13 | PDE_03453- PDE_03455 | PKS (PDE_03455) |  |
| Cluster_14 | PDE_03920- PDE_03926 | PKS (PDE_03926) |  |
| Cluster_15 | PDE_04008- PDE_04024 | HYBRID (PDE_04017)  PKS (PDE_04018) | Yes |
| Cluster_16 | PDE_04252- PDE_04267 | HYBRID (PDE_04252) |  |
| Cluster_17 | PDE_04540 - PDE_04545 | NRPS (PDE_04253) |  |
| Cluster_18 | PDE_05556 - PDE_05569 | NRPS (PDE_05564) |  |
| Cluster_19 | PDE_06202 - PDE_06213 | NRPS-Like (PDE_02203) |  |
| Cluster_20 | PDE_06623 - PDE_06230 | NRPS-Like (PDE_06630) | Yes |
| Cluster_21 | PDE_07001 - PDE_07006 | NRPS (PDE_07005) | Yes |
| Cluster_22 | PDE_07162 - PDE_07172 | NRPS (PDE_07173) |  |
| Cluster_23 | PDE_07373 - PDE_07378 | NRPS-Like (PDE_07373) |  |
| Cluster_24 | PDE_08154 - PDE_08155 | NRPS-Like (PDE_08155) |  |
| Cluster_25 | PDE_09188 - PDE_09202 | HYBRID (PDE_09198) | Yes |
| Cluster_26 | PDE_09227 - PDE_09243 | PKS (PDE_09237) | Yes |
| Cluster_27 | PDE_09491 - PDE_09496 | PKS (PDE_09491) | Yes |
| Cluster_28 | PDE_09499 - PDE_10009 | PKS (PDE_10006) | Yes |

^a^: DMAT, Demethylallyl tryptophan synthase; NRPS, Nonribosomal peptide synthetases; PKS, lyketide synthases; HYBRID, PKS-NRPS hybrid.
