# Supplementary material for: F-box protein Fbx23 acts as a transcriptional coactivator to recognize and activate transcription factor Ace1
Source: PLoS Genet. 2025 Jan 21;21(1):e1011539. doi: 10.1371/journal.pgen.1011539 (PMC11750091; doi:10.1371/journal.pgen.1011539)
Supplement: S2 Table — (DOCX) [file pgen.1011539.s003.docx]

**Table S2 Primers used in this study**

| Primers | Primer sequences (5'-3') |
| --- | --- |
| Construction of Ace1/Fbx23-TAP strain | |
| Ace1-TAP-UF | CTCGTCCACTCTAGAACAATCG |
| Ace1-TAP-UR | CTTGTCGTCGTCGTCCTTGTAGTCATCGAACGCGATGGCTTTGTCC |
| Ace1-TAP-DF | CGTCACCAGCCCCTGGGTTGGGTATTGTTCGTCGATGAAC |
| Ace1-TAP-DR | CTCCTTCTCTTGAATTGGCACTG |
| Ace1-TAP-CSF | GGCCCAGCACTTCCCATCATC |
| Ace1-TAP-CSR | CTTCACTTGCACTGATTATCTGG |
| Fbx23-TAP-UF | GCAAACTGGCACCATGGTCG |
| Fbx23-TAP-UR | CTTGTCGTCGTCGTCCTTGTAGTCAAGTCCTTGGAAGAAACGACATG |
| Fbx23-TAP-DF | CGTCACCAGCCCCTGGGTTGTCTTCAGTTCTACGATGTGATTTG |
| Fbx23-TAP-DR | CAACGTGAGACCTGGCTTCG |
| Fbx23-TAP-CSF | GCCCAGAGGAGGACATCATC |
| Fbx23-TAP-CSR | GCCTCATTTTCAGGAGCGAC |
| hph-F | CGACGTTAACTGATATTGAAGGA |
| hph-R | CAACCCAGGGGCTGGTGACGGAA |
| hph-YZF | GTACTCGCCGATAGTGGAAACCG |
| hph-YZR | CTGCTAATAAGAGTCACACTTC |
| Construction of Ace1/Fbx23-GFP strain | |
| Ace1-GFP-UR | CAGCTCCTCGCCCTTGCTCACCATTCCTCCTCCTCCATCGAACGCGATGGCTTTGTC |
| fbx23-GFP-UF | GCAAACTGGCACCATGGTCG |
| fbx23-GFP-UR | CAGCTCCTCGCCCTTGCTCACCATTCCTCCTCCTCCAAGTCCTTGGAAGAAACGAC |
| fbx23-GFP-CSF | CACTAGAGTGGCCGAAACCCTG |
| gfp-F | ATGGTGAGCAAGGGCGAGGAGCTGT |
| gfp-R | TTCAATATCAGTTAACGTCGTTACTTGTACAGCTCGTCCATGCCG |
| Construction and verification of the Δ*fbx23*, Re*fbx23*, and ΔF-Ace1-GFP | |
| Δfbx23-UF | GGAAGAAAGGGACAGTCGCG |
| Δfbx23-UR | CCTTCAATATCAGTTAACGTCGCCGCGCGGGCGACGATCCCG |
| Δfbx23-DF | CGTCACCAGCCCCTGGGTTGTCTTCAGTTCTACGATGTGATTTG |
| Δfbx23-DR | CAACGTGAGACCTGGCTTCG |
| Δfbx23-CSF | CACTAGAGTGGCCGAAACCCTG |
| Δfbx23-CSR | GCCTCATTTTCAGGAGCGAC |
| Δfbx23-YZF | GAATCCACTGTCCTCCCCTC |
| Δfbx23-YZR | GATACACACTGTCGAGAGTGG |
| fbx23-sou-F | CACGAGAGAATCACGCCGAATC |
| fbx23-sou-R | GACCACAACAAACGGCACGTC |
| ptrA-F | GGGCAATTGATTACGGGATCCCA |
| ptrA-R | CCACCGTTACATACGGGACACAG |
| Refbx23-UF | GGAAGAAAGGGACAGTCGCG |
| Refbx23-UR | GATCCCGTAATCAATTGCCCTTAAAGTCCTTGGAAGAAACGAC |
| ptrA-YZF | CTAGGATCCTAGTCTAGAAG |
| ptrA-YZR | ATCTGGGGGTTGCCGTTCTC |
| Δfbx23(Ace1-GFP)-UR | GGATCCCGTAATCAATTGCCCCCGCGCGGGCGACGATCCCGG |
| Δfbx23(Ace1-GFP)-DF | GAGCGGCTCATCGTCACCCCATTCTTCAGTTCTACGATGTGAT |
| Construction of the BiFC strain | |
| Ace1-NF | CAACCTTCACCACCTCGAGCTCGCTAGCATGGCGTCTACACAATTTGAG |
| Ace1-NR | GATGGAGCGGCGGCCGCTGTGGGTACCATCGAACGCGATGGCTTTGTC |
| fbx23-CF | CATTCTTACGACTGCCGTCAGCGGCCGCATGGAGGAACAGACTGGACC |
| fbx23-CR | CTTGCAGGCCGGGCGCCTAGGGTCGACAAGTCCTTGGAAGAAACGAC |
| NYZF | TTGGGTTGTACGATCACCAACAC |
| NYZR | GAGATAGAGAATTGTGTGGGATGAG |
| CYZF | GTCAAGAGACCTACGAGACTGAG |
| CYZR | CGTGAACAAGGGACATTACAATG |
| Construction of the Y2H strain | |
| A396-ADF | GCTCATATGGCCATGGAGGCCGCGTCTACACAATTTGAGTCG |
| A396-ADR | TCATCTGCAGCTCGAGCTCGGTTGATGTCCATGGGCGGTG |
| A816-ADF | GCTCATATGGCCATGGAGGCCGGATGGAAGTACAACCGTCAG |
| A816-ADR | TCATCTGCAGCTCGAGCTCGCTAATCGAACGCGATGGCTTTGTC |
| Fbx23-BDF | GACCTGCATATGGCCATGGAGGAGGAACAGACTGGACCAGACTC |
| Fbx23-BDR | CTAGTTATGCGGCCGCTGCAGTTAAAGTCCTTGGAAGAAACGAC |
| YZF | TAATACGACTCACTATAGGGC |
| ADYZR | CATGGCCAAGATTGAAACTTAGAG |
| BDYZR | GCTATGACCATGATTACGCC |
| For RT-qPCR | |
| Actin-RT-F | GTTCCATTCTCGCCTCCCTCT |
| Actin-RT-R | AGAAGCACTTGCGGTGAACGA |
| cbh1-RT-F | CCACCACCACTACCAGCAAGG |
| cbh1-RT-R | GTAGCCAACACCACCGCACT |
| xlnR-RT-F | CGATCCGCTCTTGCCCAGGTA |
| xlnR-RT-R | GGGCGAGAACTTCACGTCTG |
| creA-RT-F | TGTCCCGCAGGTCCCTAAAGT |
| creA-RT-R | GCCCGCCACGGAATTATTTGT |
| eg1-RT-F | CCACCACCAAGATTTCCACC |
| eg1-RT-R | GGACACGCAGGCTGTAGGTC |
| clrB-RT-F | CCATCGAATCTTGCCAAGCAC |
| clrB-RT-R | GCTTGCTGGCTTCGTAAATGC |
| Ace1-RT-F | CGCGGCCAACATTGGCGCCTCTTTC |
| Ace1-RT-R | CTAATCGAACGCGATGGCTTTGTC |
| brlA-RT-F | CCGATTACGATCCTGAGTTCCGAG |
| brlA-RT-R | CTAGTCCTCCCAGCCATCCACG |
| abr2-RT-F | GACCCACTTGAGCAACGGCATGG |
| abr2-RT-R | TCAGTTTCTATGGGCACGGG |
| abr1-RT-F | TGGATCCTGGCCTGAGTCGTC |
| abr1-RT-R | CGTTATCATGGGTCTGAATG |
| ayg1-RT-F | GAGGGTTCGGTCCACATGGGC |
| ayg1-RT-R | CTAGTTCTTGACCTCTGCCGTG |
| arp1-RT-F | CTACTATCGCAAGGTGGATGG |
| arp1-RT-R | TCAATCCAGCCCCTTGAATAC |
| arp2-RT-F | CTTGAAGCGATGTGCCGTTCC |
| arp2-RT-R | TCAGATGTTAGCCCCTCCGTTC |
| alb1-RT-F | GCATCACAGTCATGGAAGAAGC |
| alb1-RT-R | TCAACAGGAGCTCATGGCACTAG |
| For Chip-qPCR |  |
| abr2-1F(R1) | CAAGCGTGGAAAGCATAGAATTG |
| abr2-1R(R1) | CCGTGTTCGCGGCAAGAGGAATG |
| abr2-2F(R2) | CACCGTTCTTCGCTCAAGGATGC |
| abr2-2R(R2) | GATCGCCCCTCCAACATCGCGG |
| abr2-3F(R3) | CTCCCATAACATTCCTCCGGAC |
| abr2-3R(R3) | GATGAGTACCTGGGCACAGGC |
| abr2-5F(R5) | CACTCTCCTGCCTCTTGTGGC |
| abr2-5R(R5) | CTAGGCGCAGCTCAGGGCCCG |
| abr2-6F(R6) | CGCACTTGATCATCCTCTCGGA |
| abr2-6R(R6) | GTGATTGCAGATACGGCAGTTC |
| brlA-1F(R1) | CAGAGGCTATTGTCTGCATTCCTG |
| brlA-1R(R1) | CGAGCAGAAATAAACAATTCGG |
| brlA-2F(R2) | CCGCCTCGCTACCTTTGGTCTC |
| brlA-2R(R2) | CACAGTGGAGGGTTGACCCGAG |
| brlA-3F(R3) | GGTCTCCGTGGTCATTCATTC |
| brlA-3R(R3) | GTCGTCGAGTTCTTCAATTTTG |
| brlA-4F(R4) | CACAAGGTCAGCAAATCTCGGAC |
| brlA-4R(R4) | CGTGGCTGTAAAGACTGGTAG |
| brlA-5F(R5) | CCTTTGAACACCCGACATTCCC |
| brlA-5R(R5) | CATGGGACGAGACAGGCACGG |
| brlA-6F(R6) | CTCCACGGAGTGACACTCCAC |
| brlA-6R(R6) | CAAGTTGGATCGCACTAATTG |
| cbh1-1F(R1) | GCGCGGGCGTGTCACATTTCG |
| cbh1-1R(R1) | GGAGCCCATGCAAATGACAATG |
| cbh1-2F(R2) | CATATCCTTCACCGAGTGGGTTG |
| cbh1-2R(R2) | GACGATGGAGGCGAAATTGAGG |
| cbh1-3F(R3) | CTGGGGAGCTTCCTCCGTGTCCG |
| cbh1-3R(R3) | CAATTCGCCTCTCGACTGATTTC |
| cbh1-4F(R4) | CTTCAGGATCCACCCAAGGAAG |
| cbh1-4R(R4) | GTGATGGATTGGATCAAAGATC |
| cbh1-5F(R5) | CTCACTCCTCACCCTTGCGACAC |
| cbh1-5R(R5) | GTAGAAGTTGGTCACACCCTG |
| eg1-1F(R1) | CTGTAGATCCTTGTCTGTTTGGC |
| eg1-1R(R1) | GATGGGGGAGATGCAGGTGGAGC |
| eg1-2F(R2) | GCAGTTCGACTGGGTGAGAAGG |
| eg1-2R(R2) | CTTTTCGCCAGGTGAGAGATG |
| eg1-3F(R3) | CAGATGCATCATCTCTCACCTG |
| eg1-3R(R3) | CCTTGGGGTGAAACTCTTCAAC |
| eg1-4F(R4) | CTTTCACAAGGAGACAGTTCCTC |
| eg1-4R(R4) | CCGACGTGTCCTGAGCAACACAG |
| eg1-5F(R5) | GTACGGTTCAGGATACTGCGACG |
| eg1-5R(R5) | GTCATCGGAGCTGCAAGGGTGAG |
| xyn11A-1F(R1) | GGTGACCACGATGTCTGAACGAG |
| xyn11A-1R(R1) | CAGAAACGAGCCTAGTCTGCAG |
| xyn11A-2F(R2) | GATAGACATGCCAAACCTACCTATC |
| xyn11A-2R(R2) | CACAGAGTGACGAATGATCGAAG |
| xyn11A-3F(R3) | CAGTGAATCCTCTTCGATCATTC |
| xyn11A-3R(R3) | CTCTGCTGGTGTACCGGTATTC |
| xyn11A-4F(R4) | CGCCGTACTCGGGCTCAGTGG |
| xyn11A-4R(R4) | CGCACTGAGCCCTGTGTGCTG |
| xyn11A-5F(R5) | CGGGTCCTAGCACCACCGCTA |
| xyn11A-5R(R5) | GGTCCAAGCACCACATGAGCTC |
