# Supplementary figures and images for: F-box protein Fbx23 acts as a transcriptional coactivator to recognize and activate transcription factor Ace1
Source: PLoS Genet. 2025 Jan 21;21(1):e1011539. doi: 10.1371/journal.pgen.1011539 (PMC11750091; doi:10.1371/journal.pgen.1011539)

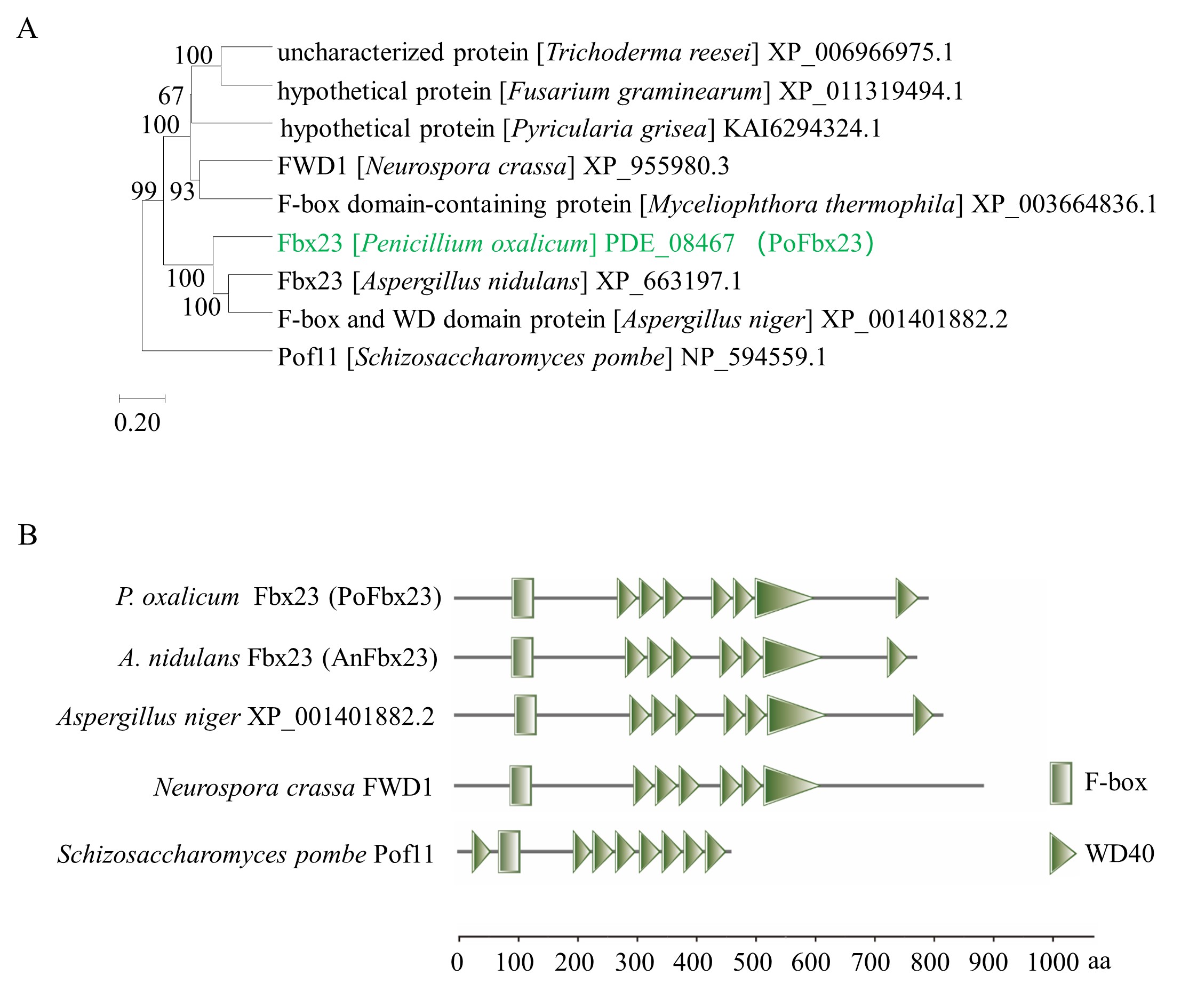

Supplement: S1 Fig — (A) Phylogenetic analysis of PoAce1 orthologs. Multiple sequence alignment was performed, and a phylogenetic tree was constructed using the maximum likelihood method by Molecular Evolutionary Genetic Analysis (MEGA 7.0). Coefficients were indicated below the respective nodes, and gaps in the alignment were not considered. (B) Domain architecture analysis refers to the website SMART (http://smart.embl-heidelberg.de/). The maps were constructed with equal proportions of the respective sequences. (JPG) [file pgen.1011539.s004.jpg]

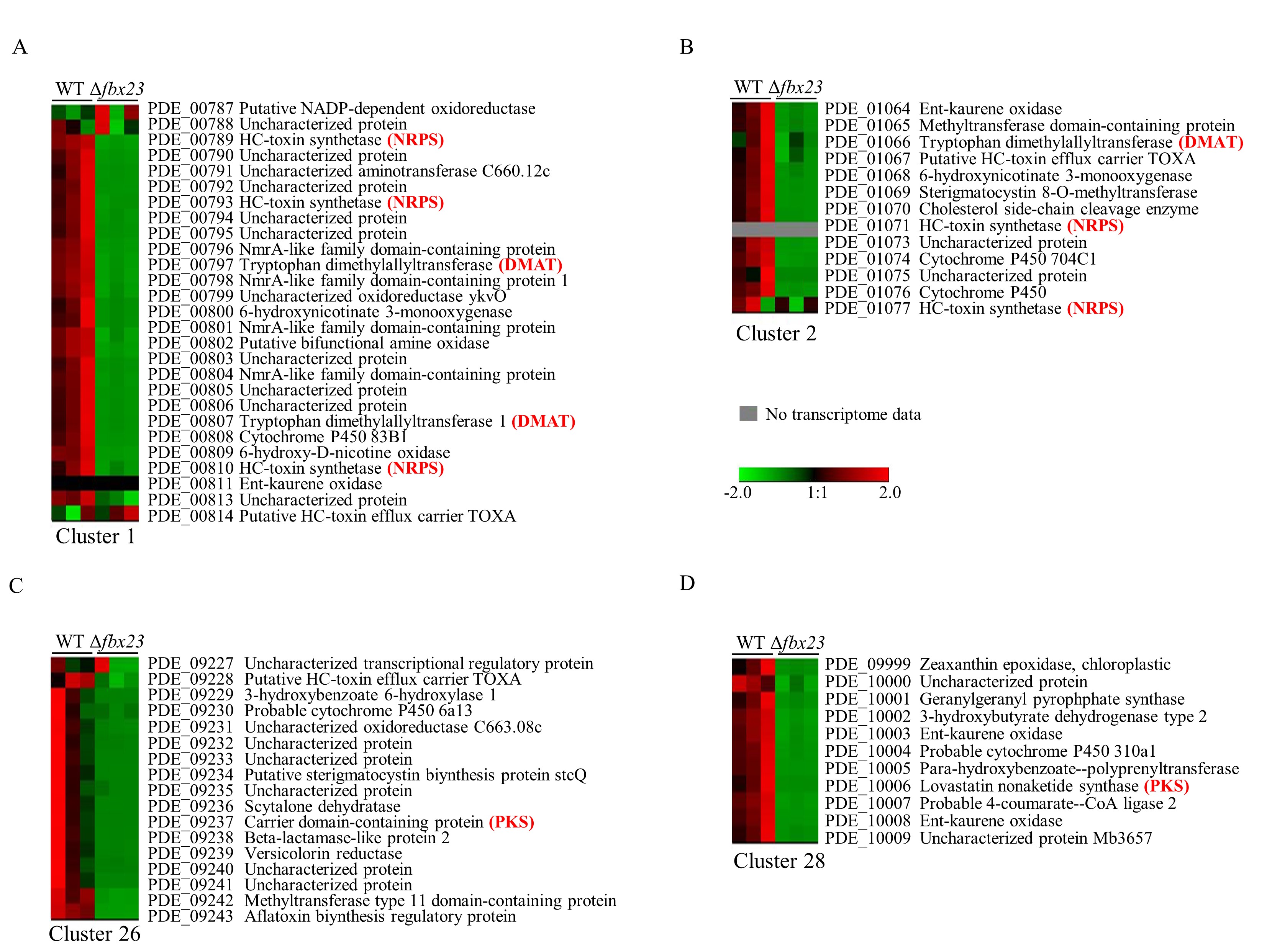

Supplement: S2 Fig — The color of each block represents the log2(fold change) in gene expression. The red fonts indicate the “backbone” genes predicted in the clusters. (A) Cluster 1 (B) Cluster 2 (C) Cluster 26 (D) Cluster 28. The description of 28 secondary metabolic gene clusters is shown in S1 Table. DMAT, Demethylallyl tryptophan synthase; NRPS, Nonribosomal peptide synthetases; PKS, Polyketide synthases; HYBRID, PKS-NRPS hybrid. (JPG) [file pgen.1011539.s005.jpg]

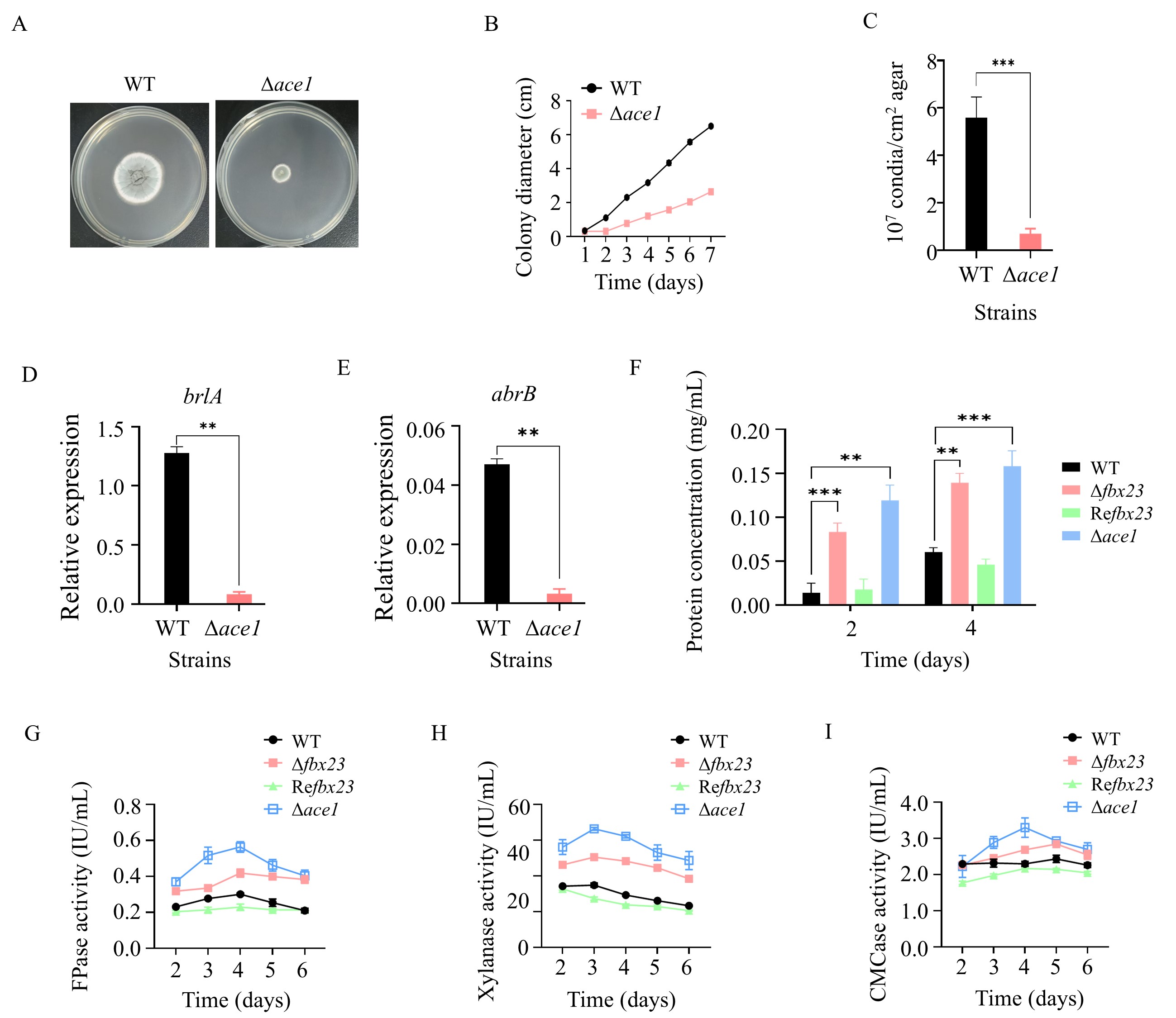

Supplement: S3 Fig — Colonies (A) and colony diameters (B) of P. oxalicum WT and the Δace1 mutant grown on VMMG agar at 30°C. (B) Conidia quantification after the WT and the Δace1 mutant grown on VMMG agar at 30°C for five days. Transcription levels of the gene brlA (D) and abrB (E) assayed by qRT-PCR after the WT and the Δace1 mutant grown in VMMG liquid at 30°C for 24 hours, respectively. Assay of extracellular protein concentration (F), FPase activity (G), Xylanase activity (H), and CMCase activity (I) of the WT the mutants grown in VMMC liquid. The biological triplicates were performed for all enzymatic activity analyses. The mean values and standard deviations were calculated. *p < 0.05, **p < 0.01, ***p < 0.001. (JPG) [file pgen.1011539.s006.jpg]

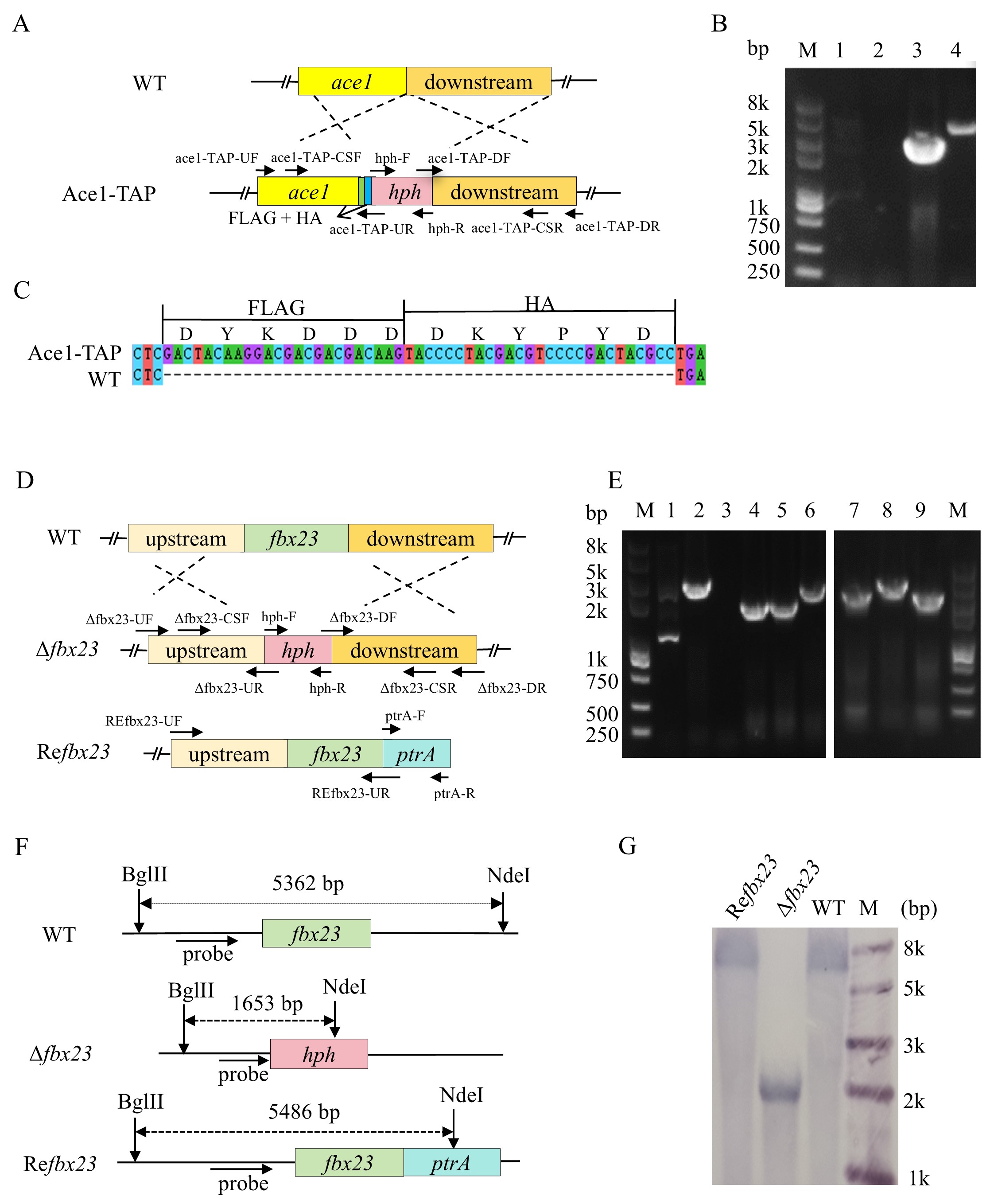

Supplement: S4 Fig — (A) Construction strategy of Ace1-TAP strain. The construction strategy of the Fbx23-TAP strain is the same as that of the Ace1-TAP strain. (B) Results of diagnostic PCR of TAP strains. Lane 1 and lane 2 represent the control P. oxalicum WT; lane 3 (2555 bp) and lane 4 (3015 bp) represent Ace1-TAP (amplified using primers ace1-TAP-UF/hph-YZR and hph-YZF/ace1-TAP-DR, respectively). (C) Sequencing results of the protein PoAce1 fused with the TAP (FALG-HA) tag. (D) Construction strategies of strains Δfbx23 and Refbx23. (E) Results of diagnostic PCR of strains Δfbx23 and Refbx23. Lanes 1, 2, and 3 represent the negative control WT; lane 4 (1964 bp). Lane 5 (2122 bp) and Lane 6 (2434 bp) represent Δfbx23 (amplified using primers Δfbx23-UF/hph-YZR, fbx23-MYZF/fbx23-MYZR and hph-YZF/Δfbx23-DR, respectively). Lane 7 (4748 bp), lane 8 (4989 bp) and lane 9 (3879 bp) represent Refbx23 (amplified using primers Δfbx23-UF/ptrA-YZR, fbx23-MYZF/fbx23-MYZR and ptrA-YZF/Δfbx23-DR, respectively). (F) Strategies of the Southern blot. Primers fbx23-sou-F/fbx23-sou-R were used to amplify the probe. (G) The results of Southern blot. The theoretical size of WT is 5362 bp; the theoretical size of the Δfbx23 mutant strain is 1653 bp; the theoretical size of Refbx23 is 5468 bp. (JPG) [file pgen.1011539.s007.jpg]

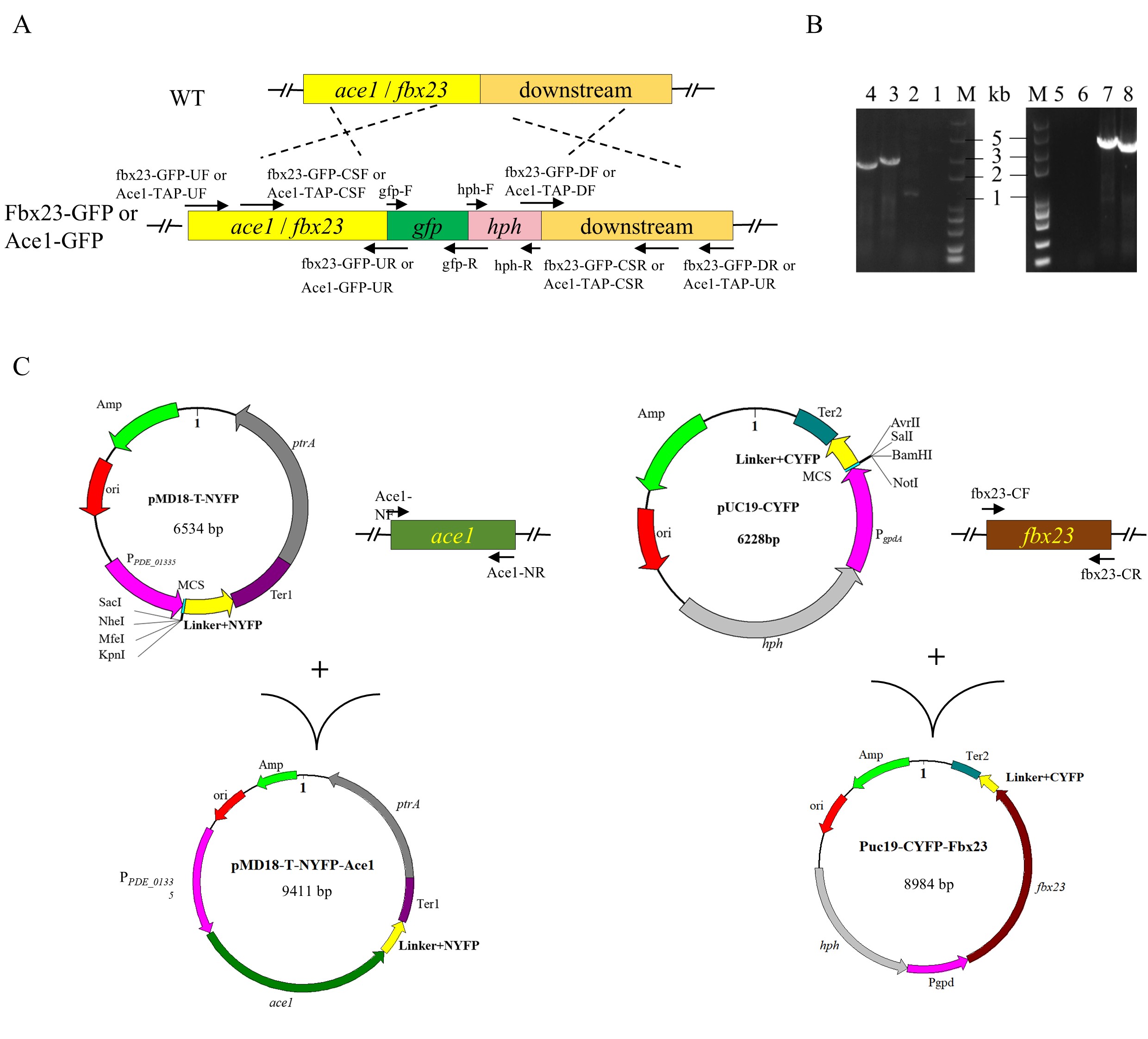

Supplement: S5 Fig — (A) Construction strategy of GFP strains Ace1-GFP and Fbx23-GFP. (B) Results of diagnostic PCR of GFP strains. Lanes 1, 2, 5 and 6 represent the control WT; lane 3 (2555 bp) and lane 4 (3015 bp) represent Ace1-GFP (amplified using primers Ace1-GFP-UF/hph-YZR and hph-YZF/Ace1-GFP-DR, respectively); lane 7 (2699 bp) and lane 8 (2434 bp) represent Ace1-GFP (amplified using primers Fbx23-GFP-UF/hph-YZR and hph-YZF/Fbx23-GFP-DR, respectively). (C) Construction strategy of BiFC strain Ace1-YFP-Fbx23. (JPG) [file pgen.1011539.s008.jpg]

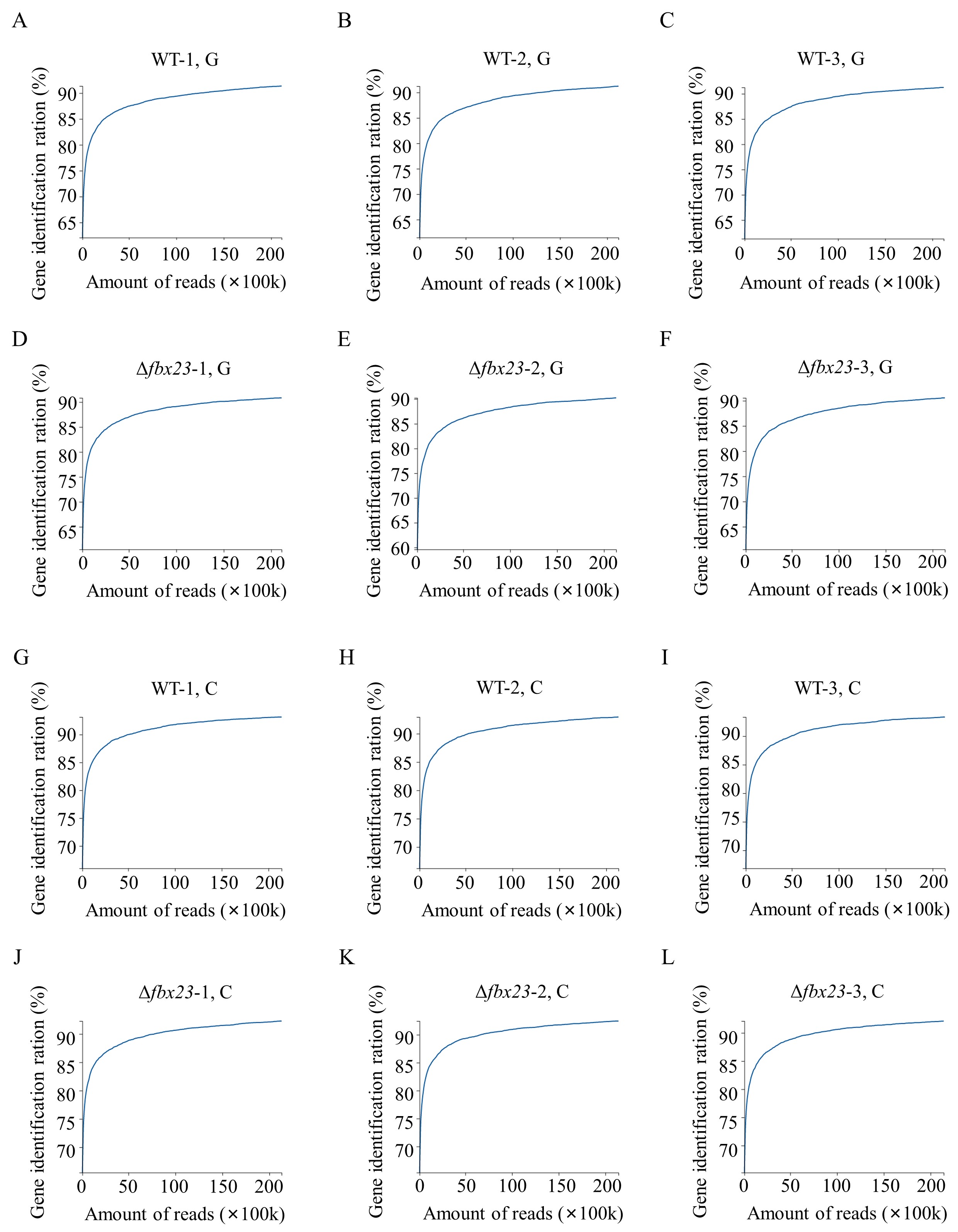

Supplement: S6 Fig — (A~C) The biological triplicates of the WT grown in VMMG. (D~F) Biological triplicates of the Δfbx23 grown in VMMG. (G~I) Biological triplicates of the WT grown in VMMC. (J~L) Biological triplicates of the Δfbx23 grown in VMMC. (JPG) [file pgen.1011539.s009.jpg]
